# mTOR pathway diseases: challenges and opportunities from bench to bedside and the mTOR node

**DOI:** 10.1186/s13023-025-03740-1

**Published:** 2025-05-27

**Authors:** Laura Mantoan Ritter, Nicholas M. P. Annear, Emma L. Baple, Leila Y. Ben-Chaabane, Istvan Bodi, Lauren Brosson, Jill E. Cadwgan, Bryn Coslett, Andrew H. Crosby, D. Mark Davies, Nicola Daykin, Stefanie Dedeurwaerdere, Christina Dühring Fenger, Elaine A. Dunlop, Frances V. Elmslie, Marie Girodengo, Sophie Hambleton, Anna C. Jansen, Simon R. Johnson, Kelly C. Kearley, John C. Kingswood, Liisi Laaniste, Katherine Lachlan, Andrew Latchford, Ralitsa R. Madsen, Sahar Mansour, Simeon R. Mihaylov, Louwai Muhammed, Claire Oliver, Tom Pepper, Lettie E. Rawlins, Ina Schim van der Loeff, Ata Siddiqui, Pooja Takhar, Katrina Tatton-Brown, Andrew R. Tee, Priyanka Tibarewal, Charlotte Tye, Sila K. Ultanir, Bart Vanhaesebroeck, Benjamin Zare, Deb K. Pal, Joseph M. Bateman

**Affiliations:** 1 King’s College London Institute of Psychiatry Psychology and Neuroscience, London, UK; 2 St George’s University Hospitals NHS Foundation Trust, London, UK; 3 University of Exeter Medical School, Exeter, UK; 4 King’s College Hospital NHS Foundation Trust, London, UK; 5 mTOR Node Advisory Panel (MAP), London, UK; 6 Guy’s and St Thomas’s NHS Foundation Trust, London, UK; 7 Amplexa Genetics, Odense, Denmark; 8 Cardiff University, Cardiff, UK; 9 UCB Biopharma SRL, Brussels, Belgium; 10 The Francis Crick Institute, London, UK; 11 Newcastle University Translational and Clinical Research Institute, Newcastle University, Newcastle upon Tyne, UK; 12 Antwerp University Hospital, Antwerp, Belgium; 13 PTEN UK and Ireland Patient Group, London, UK; 14 Centre for Respiratory Research, NIHR Nottingham Biomedical Research Centre and Biodiscovery Institute, Translational Medical Sciences, University of Nottingham, Nottingham, UK; 15 University Hospital Southampton NHS Foundation Trust, Southampton, UK; 16 CoSyne Therapeutics, London, UK; 17 Polyposis Registry, St Mark’s Hospital, London, UK; 18 University of Dundee School of Life Sciences, Dundee, UK; 19 PTEN Research, Cheltenham, Gloucestershire UK; 20 Tuberous Sclerosis Association, London, UK; 21 UCL Cancer Institute, London, UK; 22 Department of Surgery and Cancer, Imperial College London, London, UK; 23 School of Health & Medical Sciences, City St George’s, University of London, London, UK

**Keywords:** mTOR, Rare diseases, Tuberous sclerosis complex, PTEN, PI3K, AKT, Everolimus, Rapamycin, Peutz-Jeghers, Birt-Hogg-Dubé

## Abstract

Mechanistic target of rapamycin (mTOR) is a highly conserved serine/threonine kinase that regulates key cellular processes including cell growth, autophagy and metabolism. Hyperactivation of the mTOR pathway causes a group of rare and ultrarare genetic diseases. mTOR pathway diseases have diverse clinical manifestations that are managed by distinct medical disciplines but share a common underlying molecular basis. There is a now a deep understanding of the molecular underpinning that regulates the mTOR pathway but effective treatments for most mTOR pathway diseases are lacking. Translating scientific knowledge into clinical applications to benefit the unmet clinical needs of patients is a major challenge common to many rare diseases. In this article we expound how mTOR pathway diseases provide an opportunity to coordinate basic and translational disease research across the group, together with industry, medical research foundations, charities and patient groups, by pooling expertise and driving progress to benefit patients. We outline the germline and somatic mutations in the mTOR pathway that cause rare diseases and summarise the prevalence, genetic basis, clinical manifestations, pathophysiology and current treatments for each disease in this group. We describe the challenges and opportunities for progress in elucidating the underlying mechanisms, improving diagnosis and prognosis, as well as the development and approval of new therapies for mTOR pathway diseases. We illustrate the crucial role of patient public involvement and engagement in rare disease and mTOR pathway disease research. Finally, we explain how the mTOR Pathway Diseases node, part of the Research Disease Research UK Platform, will address these challenges to improve the understanding, diagnosis and treatment of mTOR pathway diseases.

## mTOR signalling as an exemplar of the challenges and opportunities in rare disease research

Affecting fewer than 1 in 2000 individuals [1], rare diseases are individually rare but collectively have a prevalence of 3.5–5.9% affecting 263–446 million people globally and therefore, when considered in aggregate, place a significant burden on effected individuals, families, and healthcare systems [2]. Compared to common diseases, the small patient populations in rare and ultrarare diseases have resulted in both historically meagre public funding for research into the underlying mechanisms and in ambivalence of industry towards drug development and pursuing clinical trials; as a consequence, only 5–15% of rare diseases have drug treatments [3]. Although rare disease research has recently gained momentum, there remain significant barriers to overcoming the challenges. These barriers stem from the small patient populations and often complex pathophysiology of rare diseases, even after the causative variant is identified. Even though patient numbers for individual conditions are small, the impact on the lives of the people with rare diseases and their support networks can be enormous. 75% of rare diseases affect children and are fatal before the age of 5 in 30% of children [4]. Progress in basic and translational research leading to benefits in the understanding, diagnosis and treatment of rare diseases will therefore have a huge impact on these patients and their communities.

**Fig. 1:**
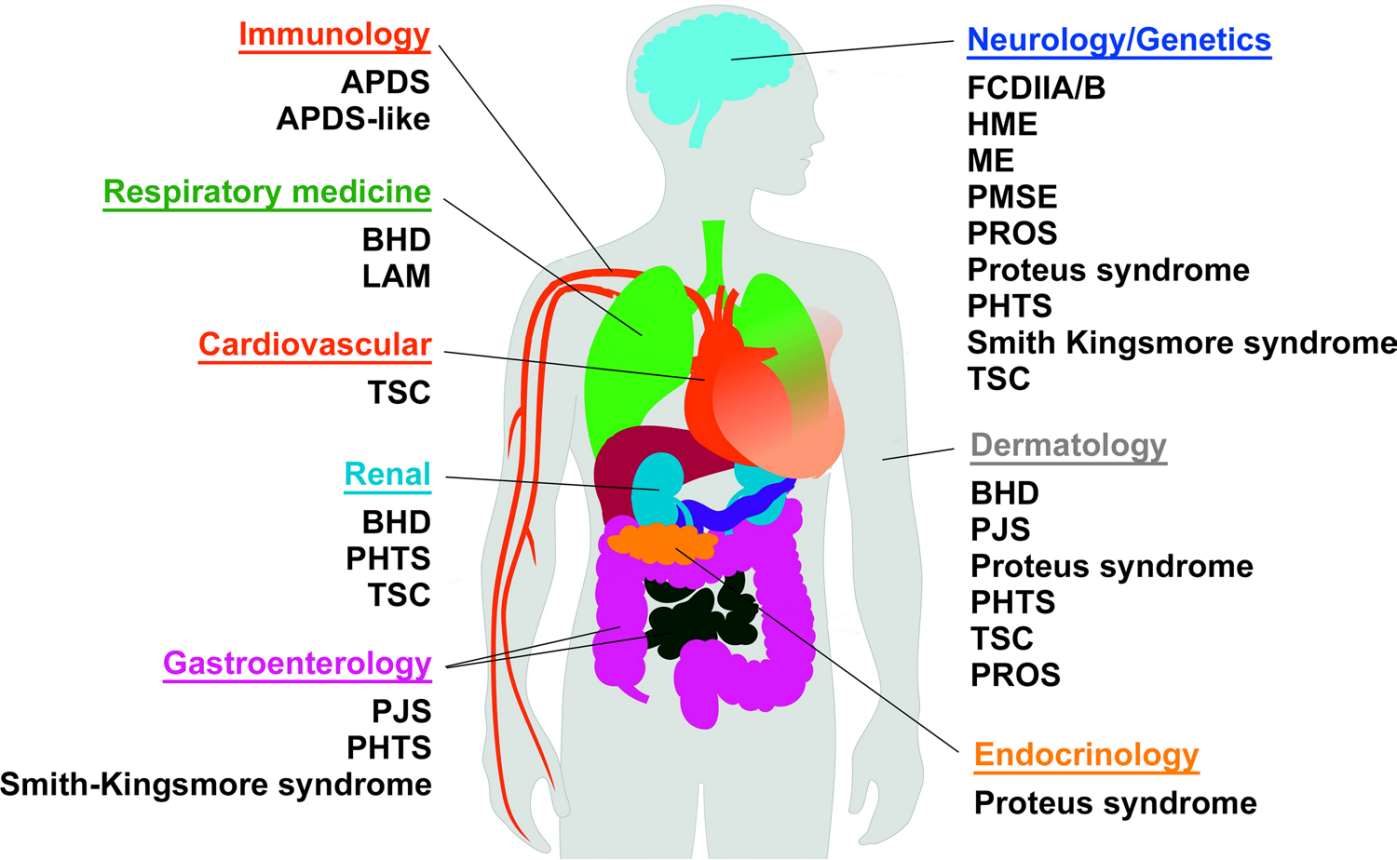
The different medical specialities that manage patients with mTOR pathway diseases. APDS: activated PI3K delta syndrome, BHD: Birt-Hogg-Dubé, FCDIIA/B: focal cortical dysplasia type IIA/B, HME: hemimegalencephaly, LAM: lymphangioleiomyomatosis, ME: megalencephaly, PMSE: polyhydramnios, megalencephaly and symptomatic epilepsy syndrome, PHTS: PTEN hamartoma tumour syndrome, PJS: Peutz-Jeghers syndrome, PROS: PIK3CA-related overgrowth spectrum, TSC: tuberous sclerosis complex

**Fig. 2:**
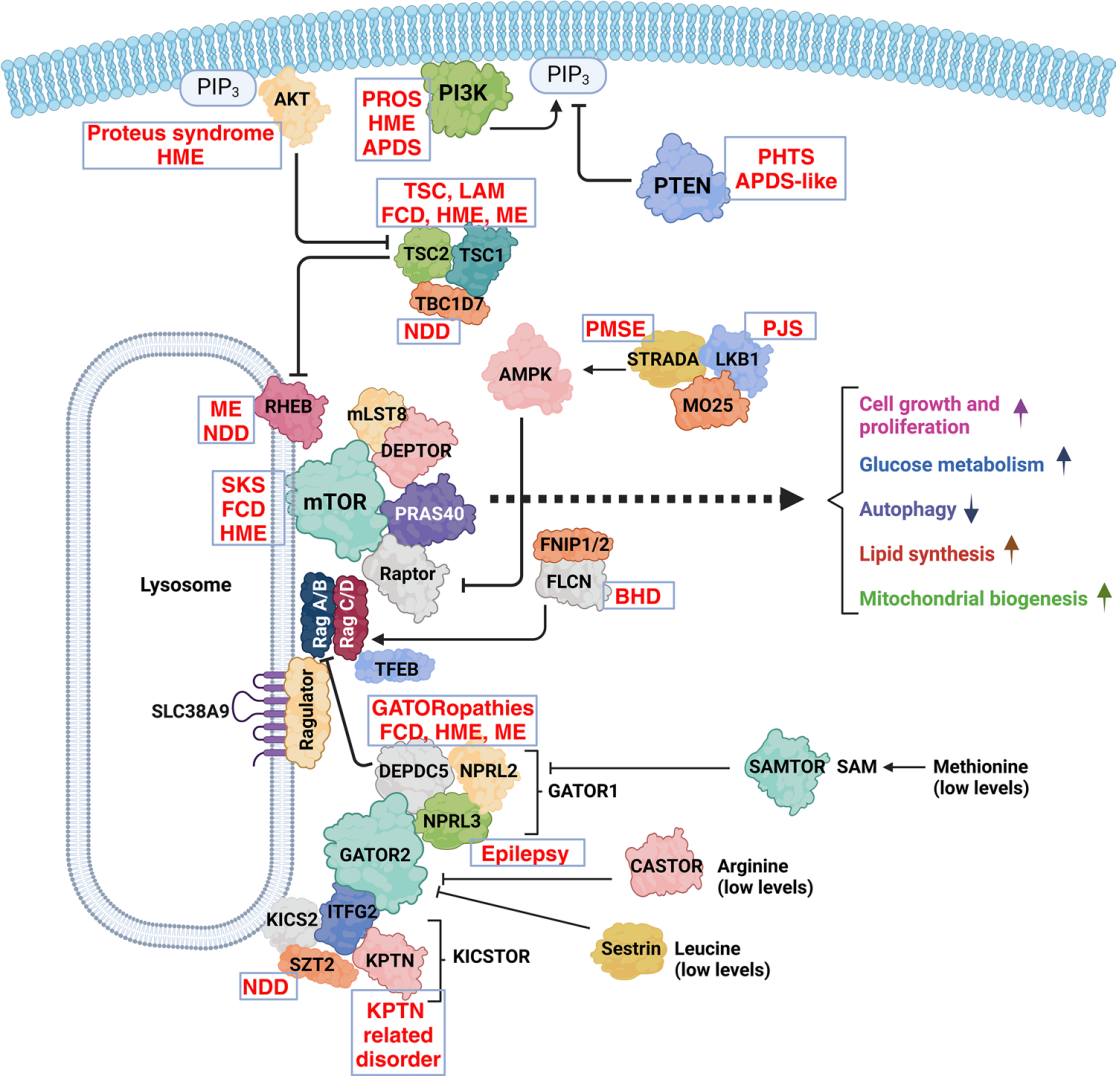
The mTOR pathway with rare diseases (shown in red) caused by mutations in specific proteins. APDS: activated PI3K delta syndrome, BHD: Birt-Hogg-Dubé, FCD: focal cortical dysplasia type IIA/B, HME: hemimegalencephaly, LAM: lymphangioleiomyomatosis, ME: megalencephaly, NDD: neurodevelopmental disease, PMSE: polyhydramnios, megalencephaly and symptomatic epilepsy syndrome, PHTS; PTEN hamartoma tumour syndrome, PJS: Peutz-Jeghers syndrome, PROS: PIK3CA-related overgrowth spectrum, SKS: Smith Kingsmore syndrome, TSC: tuberous sclerosis complex

The mechanistic target of rapamycin (mTOR) pathway diseases (Table 1) is a group of rare early-onset, hard-to-treat genetic diseases with symptoms ranging from benign tumours in multiple organs to brain malformations causing epilepsy, each of which is managed in disconnected medical disciplines (Fig. 1). The mTOR pathway has a multitude of direct and indirect associations with numerous diseases but we only include diseases caused by mutations in core components of the mTOR pathway (Fig. 2), regardless of the affected cell types and organs or symptoms. mTOR pathway diseases share a common underlying mechanism: hyperactivation of mTOR complex 1 (mTORC1) activity (Fig. 2). Since they share common molecular mechanisms and drug targets, there is an opportunity to improve diagnosis and outcomes for mTOR pathway diseases by connecting disjointed populations, basic and translational research resources with clinical, patient and industry stakeholders. In this review, we describe mTOR pathway diseases as an exemplar highlighting the challenges and opportunities in rare and ultrarare disease research. We set out how we are tackling these research challenges and exploiting opportunities through the mTOR Pathway Diseases node [5], part of the National Institute for Health and Care Research (NIHR)/Medical Research Council (MRC) Rare Disease Research UK (RDR UK) Platform [6]. Over 5 years, the mTOR Pathway diseases node aims to transform the mechanistic understanding, diagnosis and treatment of mTOR pathway diseases.

**Table 1:** mTOR pathway diseases

mTOR pathway disease	Prevalence where known	Gene	Germline vs. somatic mutations	Main affected organs	Current treatments (approved and off-label)
Activated PI3K delta syndrome (APDS)/Activated PI3K delta syndrome like (APDS-like)		*PIK3CD/PIK3R1/PTEN*	Germline	Leukocytes	mTOR inhibitors e.g. sirolimus; PI3Kδ inhibitors e.g. leniolisib
Birt-Hogg-Dubé syndrome (BHD)	Approx. 1/200,000	*FLCN*	Germline	Skin, lungs, kidneys	N/A
Focal Cortical Dysplasia type IIA/B (FCD IIA/B) (Epilepsy)		*AKT3, PIK3CA, RHEB, MTOR, DEPDC5, TSC1/2, NPRL2, NPRL3*	Somatic	Brain	mTOR inhibitors e.g. everolimus, sirolimus
GATORopathies		*DEPDC5, NPRL2, NPRL3*	Germline	Brain	
Hemimegalencephaly/megalencephaly		*AKT3, PIK3CA, RHEB, MTOR, DEPDC5, TSC1/2, NPRL2, NPRL3*	Somatic	Brain	Hemispherotomy/functional hemispherotomy/anatomical hemispherotomy
KPTN-related disorder		*KPTN*	Germline	Brain	
Lymphangioleiomyomatosis (LAM)	Approx. 20/1,000,000	*TSC1, TSC2*	Predominately somatic/some germline cases	Lung	mTOR inhibitors e.g. sirolimus
Peutz-Jeghers Syndrome (PJS)	Approx. 1/25,000 and 1/300,000	*STK11/LKB1*	Germline	Gastrointestinal tract, mucocutaneous regions	Surveillance, polypectomy, mTOR inhibitors
PIK3CA-Related Overgrowth Spectrum (PROS)		*PIK3CA*	Predominately somatic/very rare germline cases	Overgrowth of various tissues	mTOR inhibitors e.g. sirolimus; PI3Kα inhibitors e.g. alpelisib
Pretzel syndrome/polyhydramnios, megalencephaly and symptomatic epilepsy syndrome (PMSE)		*STRADA*	Germline	Brain	
Proteus Syndrome	Approx. 200 cases worldwide	*AKT1*	Somatic	Multiple organs	AKT inhibitors e.g. miransertib
PTEN Hamartoma Tumour Syndrome (PHTS)	1/200,000–1/250,000 for Cowden syndrome	*PTEN*	Germline	Multiple organs	mTOR inhibitors e.g. everolimus, sirolimus
RHEB-associated neurodevelopmental disorder		*RHEB*	Germline		
Smith-Kingsmore Syndrome		*MTOR*	Germline	Brain	
TBC1D7-associated neurodevelopmental disorder		*TBC1D7*	Germline	Brain	
Tuberous Sclerosis Complex (TSC)	Approx. 1/6,000–1/10,000	*TSC1, TSC2*	Germline, approx. 10% mosaic	Brain, kidney, lung, heart, eyes	mTOR inhibitors e.g. everolimus, sirolimus

For details see Sect. “Rare genetic diseases caused by germline mutations in mTOR pathway genes”

## mTOR signalling

mTOR is a large (289 kDa), highly conserved serine/threonine protein kinase originally identified in the yeast *S. cerevisiae* [7–10]. mTOR regulates critical cellular processes such as cell growth, autophagy, lipid synthesis, glucose metabolism, cytoskeletal dynamics and cell survival (Fig. 2). Given its crucial cellular roles, it is not surprising that mTOR has also been associated with ageing and human diseases including metabolic disorders, neurodegeneration and cancer [11, 12].

mTOR acts as the catalytic core of two protein complexes: mTOR complex 1 (mTORC1) and mTOR complex 2 (mTORC2). While both complexes exist as homodimers resembling hollow “lozenges”, they are structurally and functionally distinct owing to their accessory proteins [11, 13, 14]. In addition to mTOR, mTORC1 is composed of regulatory-associated protein of mammalian target of rapamycin (Raptor) and mammalian lethal with SEC13 protein 8 (mLST8) (Fig. 2). Raptor is a scaffold protein and the defining subunit of the complex that senses stimuli, and recruits’ substrates, thereby conferring substrate specificity, and directs subcellular localisation of mTORC1 [11, 15]. mLST8 stabilises the interaction of mTOR and Raptor and, while it aids the activity of the complex, it appears to be non-essential as shown in mouse knockout models [16, 17]. Raptor also interacts with an inhibitory subunit of the complex, proline-rich Akt substrate of 40 kDa (PRAS40) (Fig. 2). Upon phosphorylation of PRAS40 by AKT, the endogenous inhibitor dissociates from mTORC1 and allows for substrate entry [18]. Similarly, DEP domain-containing mTOR-interacting protein (DEPTOR) is an endogenous inhibitor of the complex (Fig. 2) [19]. In addition, the drug rapamycin binds to the 12-kDa FK506-binding protein (FKBP12) and inhibits, albeit only partially [20], the activity of mTORC1 through interaction with the FKBP12-rapamycin binding (FRB) domain [13].

mTORC2 is defined by the presence of the scaffold protein rapamycin-insensitive companion of mTOR (Rictor) that directly interacts with mammalian stress-activated protein kinase-interacting protein 1 (mSin1) conferring substrate selectivity [21]. mSin1 bridges Rictor with mLST8 and, unlike with mTORC1, mLST8 is essential for the activity of mTORC2. This spatial conformation appears to prevent the binding of FKBP12, resulting in the characteristic resistance of mTORC2 to acute rapamycin treatment [22]. In addition, Rictor interacts with protein observed with Rictor-1 and 2 (Protor-1/2). Their biological role is largely elusive, but evidence suggests that Protor-1 is important for the phosphorylation of SGK1 (an mTORC2 substrate) in the kidney [23, 24]. Finally, mTORC2 also interacts with the endogenous inhibitor DEPTOR. The distinct composition of mTORC1 and mTORC2 results in the involvement and regulation of different cellular processes, with mTORC2 mediating cell survival and cytoskeletal dynamics in response to growth factors [11, 12].

mTORC1 is a crossroads for other cellular signalling pathways. Activity of mTORC1 is affected by information on extra- and intracellular states including amino acids, growth factors and ATP (Fig. 2) [11]. Many pathways for sensing and interpretation of these cues ultimately converge onto two distinct signalling axes represented by the small G proteins ras homolog enriched in the brain (RHEB) and the family of Rab GTPases, both of which are activators of mTORC1 (Fig. 2). Phosphoinositide 3-kinase (PI3K) and PTEN (phosphatase and tensin homologue) act downstream of receptor tyrosine kinases to regulate mTORC1 via RHEB (Fig. 2). The master energy sensing kinase AMPK negatively regulates mTORC1 activity through phosphorylation of Raptor [25] (Fig. 2). The LKB1-STRAD-MO25 complex acts directly upstream of AMPK, regulating activity though the kinase activity of LKB1 towards AMPK [26] (Fig. 2). Sensing the availability of growth factors and cellular energy results in the inhibition of the tuberous sclerosis complex (TSC), made up of TSC1 (hamartin), TSC2 (tuberin) and TBC1D7, leading to activation of RHEB and mTORC1.

mTORC1 activity is also influenced by the presence of amino acids, independent of the TSC, though the activity of the GATOR1 complex. GATOR1 is composed of three proteins: DEPDC5, NPRL2 and NPRL3. DEPDC5 contains a RagA-binding domain that allows it to perform its GTPase-activating protein (GAP) activity on the small GTPase RagA, thereby inactivating it (Fig. 2) [27]. RagA is a binding partner of RAPTOR and, when activated, prevents translocation of mTORC1 to the lysosome where mTORC1 can be activated by RHEB. In the presence of amino acids, RagA inhibition by GATOR1 promotes mTORC1 activity. GATOR1 activity depends on three upstream regulators: the methionine sensor SAMTOR, which binds to and inhibits GATOR1 in the absence of methionine, the GATOR2 complex and the KICSTOR complex (Fig. 2). The GATOR2 complex, consisting of the proteins WDR24, MIOS, WDR59, SEH1L and SEC13, integrates information from the arginine sensor CASTOR1 and leucine sensor Sestrin1/2 [28]. In the presence of arginine and leucine, CASTOR1 and Sestrin1/2 lose their ability to bind to and inhibit GATOR2, allowing GATOR2 to bind and inhibit GATOR1, ultimately resulting in increased mTORC1 activity (Fig. 2) [29]. The KICSTOR complex, which comprises four proteins: KPTN, ITFG2, KICS2 (previously C12ORF66) and SZT2, recruits GATOR1 to the lysosomal surface and is necessary for GATOR1 interaction with its substrates and thus acts as a negative regulator of mTORC1 activity alongside GATOR1 (Fig. 2) [30]. mTORC1 activity at the lysosome is also regulated by folliculin (FLCN) which, through its GAP activity towards RagC/D, recruits mTORC1 to the lysosomal surface (Fig. 2) [31].

mTORC1 acts as a master regulator of cell growth, proliferation and metabolism by controlling an array of cellular processes. These include important pathways such as protein and lipid synthesis, glycolysis, autophagy, mitochondrial biogenesis and maintenance (Fig. 2) [32]. Representative examples of downstream effectors of mTORC1 and their involvement in specific cellular pathways are summarised in Table 2. mTORC1 signalling has wide ranging roles at the tissue, organ and system level including skeletal muscle and adipose tissue, the adaptive immune response and nervous system development [11, 33–37].

**Table 2:** Representative examples of downstream effectors of mTORC1 and their involvement in specific cellular pathways

Downstream effector	Cellular pathway	Effect of mTORC1 activity on cellular process	References
4E-BP1 (Eukaryotic translation initiation factor 4E-binding protein 1)	Protein synthesis	Activation	[311–313]
S6K1 (Ribosomal protein S6 kinase 1)	Protein/lipid/nucleotide synthesis	Activation	[314, 315]
MYC (Myelocytomatosis oncogene)	Protein synthesis	Activation	[316]
SREBP (Sterol regulatory element-binding protein) (through inhibition of Lipin-1)	Lipid synthesis	Activation	[317]
PPARγ (Peroxisome proliferator-activated receptor gamma)	Lipid synthesis	Activation	[318]
ATF4 (Activating transcription factor 4)	Nucleotide synthesis	Activation	[319]
CAD (Carbamoyl-​phosphate synthetase 2, aspartate transcarbamoylase, dihydroorotase)	Nucleotide synthesis	Activation	[271]
HIF1α (Hypoxia inducible factor 1α)	Glycolysis	Activation	[320]
PGC1α (PPARγ coactivator 1α)	Mitochondrial biogenesis and maintenance	Activation	[321, 322]
ULK1 (Unc-51 Like Autophagy Activating Kinase 1)	Autophagy	Inhibition	[323]
ATG13 (Autophagy Related 13)	Autophagy	Inhibition	[324, 325]
UVRAG (UV radiation resistance-associated gene protein)	Autophagy	Inhibition	[326]
TFEB (Transcription Factor EB)	Lysosome biogenesis	Inhibition	[327, 328]
TFE3 (Transcription Factor E3)	Lysosome biogenesis	Inhibition	[328]

For details see Sect. “mTOR signalling”

## Rare genetic diseases caused by germline mutations in mTOR pathway genes

### Activated PI3Kdelta syndrome

Class I PI3Ks are important upstream effectors of mTOR signalling. They are heterodimeric proteins consisting of a catalytic and a regulatory subunit and are classified according to similarities in structure and function [38, 39]. The regulatory subunit is important for recruitment of the catalytic subunit to the plasma membrane, its protection from proteolytic degradation and restraint of its enzymatic activity. In response to activation of various cell surface receptors, class I PI3Ks act upon phosphatidylinositol-4,5-bisphosphate [PI(4,5)P_2_], adding another phosphate on the third position of the inositol ring to generate phosphatidylinositol-3,4,5-trisphosphate [PI(3,4,5)P_3_] [40]. PI(3,4,5)P_3_ is a second messenger and recruits AKT to the cell membrane where AKT can become fully activated leading to phosphorylation of TSC2 and activation of mTORC1 (Fig. 2). PI3Kdelta is a catalytic class I PI3K subunit predominantly expressed by leukocytes and encoded by *PI3KCD* [38, 40]. Variants in both this catalytic as well as the p85alpha regulatory subunit have been described to cause inborn errors of immunity (reviewed in [40]). Biallelic loss-of-function variants in *PI3KCD* [41–43] and *PI3KR1* (encoding p85alpha, which led to severely reduced PI3Kdelta levels) [44]), cause severe B cell lymphopenia and recurrent infections, reflecting the importance of PI3K signalling downstream of the B cell receptor. Interestingly, heterozygous *activating* variants in *PI3KCD*, are also associated with hypogammaglobulinemia and frequent airway infections [45, 46]. Two groups independently described what is now known as activated PI3Kdelta syndrome (APDS), an inborn error of the immune system (IEI) characterised by recurrent sinopulmonary infections, bronchiectasis, lymphoproliferative disease, increased risk of lymphoma and immune dysregulation [45, 46]. Heterozygous variants in *PI3KR1* leading to exon skipping and PI3K hyperactivation produce a very similar clinical phenotype (APDS2) [47–49]. T cells from patients with APDS show increased S6 phosphorylation and glucose uptake, key targets of mTOR signalling [46]. The lymphoproliferation in patients with APDS shows the best response to mTOR inhibition by sirolimus [49].

An alternative to mTOR inhibition treatment for APDS is leniolisib, a specific PI3Kdelta inhibitor repurposed from oncology that is well tolerated and might target the immune dysregulatory sequelae of APDS better than sirolimus [50]. The immunological phenotype of APDS is correctable by allogeneic haematopoietic stem cell transplant (HSCT) and a recent large cohort study showed overall survival rates of 86% with no difference between APDS 1 and 2, donor type or conditioning intensity [51].

### PTEN Hamartoma tumour syndrome and APDS-like immunodeficiency

PTEN is a lipid phosphatase that antagonises PI3K activity by converting PI(3,4,5)P_3_ and its degradation product PI(3,4)P_2_ to PI(4,5)P_2_ and PI(4)P, respectively, in the cell membrane and thus indirectly inhibiting mTORC1 pathway activity (Fig. 2) [52–55]. PTEN Hamartoma Tumour Syndrome (PHTS) is an autosomal dominant tumour predisposition syndrome resulting from whole exonic deletions, truncating, splicing, missense or promoter mutations with diverse functional effects on PTEN including haploinsufficiency, lost or reduced phosphatase activity, dominant-negative and aberrant function and/or localisation [56–59]. 11–48% of *PTEN* mutations in PHTS are de novo [60]. Genotype–phenotype correlations are not robust enough to facilitate personalisation of prognostic or screening advice [61–63]. PHTS incorporates historically described clinical syndromes including Cowden syndrome, Bannayan-Riley-Ruvalcaba syndrome, PTEN related Proteus syndrome and Proteus like syndrome, with variable expression and age-related penetrance [64–68]. The prevalence of Cowden syndrome has been estimated at 1 in 200,000–250,000 [69], but the prevalence of PHTS is unknown.

**Fig. 3:**
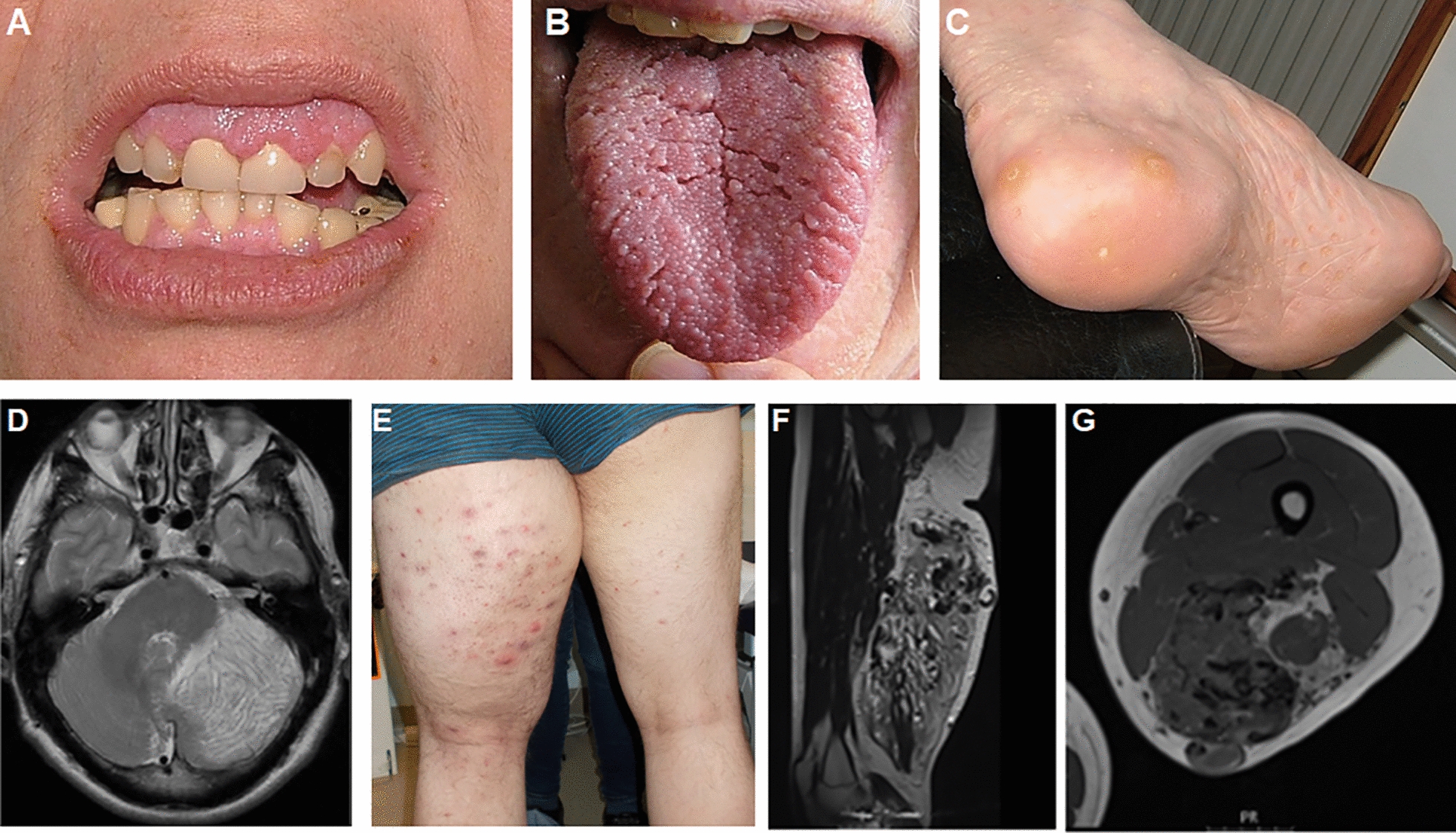
PHTS clinical manifestations. **A** Oral mucosal features. Gingival papillomatosis and neuromas may encroach on the dentition and cause difficulty with dental hygiene. **B** Papillomatosis of the tongue. **C** Plantar and palmar keratoses. Some are hyperkeratotic and may resemble viral verrucous lesions. **D** Axial T2 MRI image showing abnormal tissue in the left cerebellar hemisphere with the characteristic tigroid appearance and apparently preserved cerebellar folia of Lhermitte-Duclos Disease. **E** Segmental overgrowth of left thigh. This presented in adolescence, with no evidence of the lesion in early childhood. **F**, **G** MRI images demonstrating vascular malformation with fast and slow flow elements infiltrating muscle and fat

Clinical manifestations of PHTS include mucocutaneous, vascular, and lipomatous lesions. Malignant and benign tumours are reported including breast, thyroid, endometrium, kidney, gastrointestinal polyposis and Lhermitte-Duclos dysplastic gangliocytoma of the cerebellum (Fig. 3) [70, 71]. Macrocephaly (over 2 standard deviations (SD) above the mean) is an almost universal feature of PHTS, averaging around + 5 SD in childhood. The paediatric phenotype includes congenital macrocephaly (> 2 SD) with or without developmental delay or intellectual disability, autism spectrum disorder (ASD), lipomas, a broad spectrum of benign polyps (ganglioneuromas, inflammatory polyps), as well as polyps with malignant potential (serrated, adenomas and juvenile type hamartomas) [72, 73]. Vascular anomalies occur in approximately 50% of PHTS patients and are associated with significant morbidity and mortality [74].

Small cohort studies and case studies have recently suggested that PHTS patients can manifest an APDS-like condition (APDS-L) and can present with recurrent respiratory tract infections, hypogammaglobulinaemia, lymphopenia, lymphoproliferation changes in B and T cell subsets and auto-immune diseases [75–78].

Clinical trials using mTORC1 inhibitors (sirolimus and everolimus) are reported to be tolerated and have some efficacy in treating cutaneous and gastrointestinal features and improving cerebellar function, as well as in treatment of vascular anomalies [79–81]. Improvement in some measures, but not reaching primary endpoint was demonstrated in a 6-month phase II, randomized, double-blinded, placebo-controlled trial of treatment with everolimus for neurocognitive symptoms [79]. In a case study, two breast cancer patients with germline PTEN mutations showed a dramatic response to monotherapy with the AKT inhibitor capivasertib [82]. Inhibition of mTORC1 may also represent an avenue for chemoprevention in PHTS – a mouse model has shown rapamycin to delay tumour development [83].

### Peutz-Jeghers syndrome

Peutz-Jeghers syndrome (PJS) is an autosomal dominant cancer syndrome with an estimated prevalence of between 1/25,000 and 1/300,000 [84]. PJS is caused by germline pathogenic variants in the tumour suppressor gene *STK11/LKB1*, with subsequent somatic inactivation of the wild-type allele then resulting in loss-of-function of the kinase activity of LKB1 [85–89]. LKB1 is a master kinase that activates multiple kinases of the AMPK subfamily [90], some of which regulate the mTORC1 pathway through phosphorylation of TSC2 and Raptor (Fig. 2), with mTORC1 dysregulation likely to be involved in hamartoma and cancer development [91, 92].

**Fig. 4:**
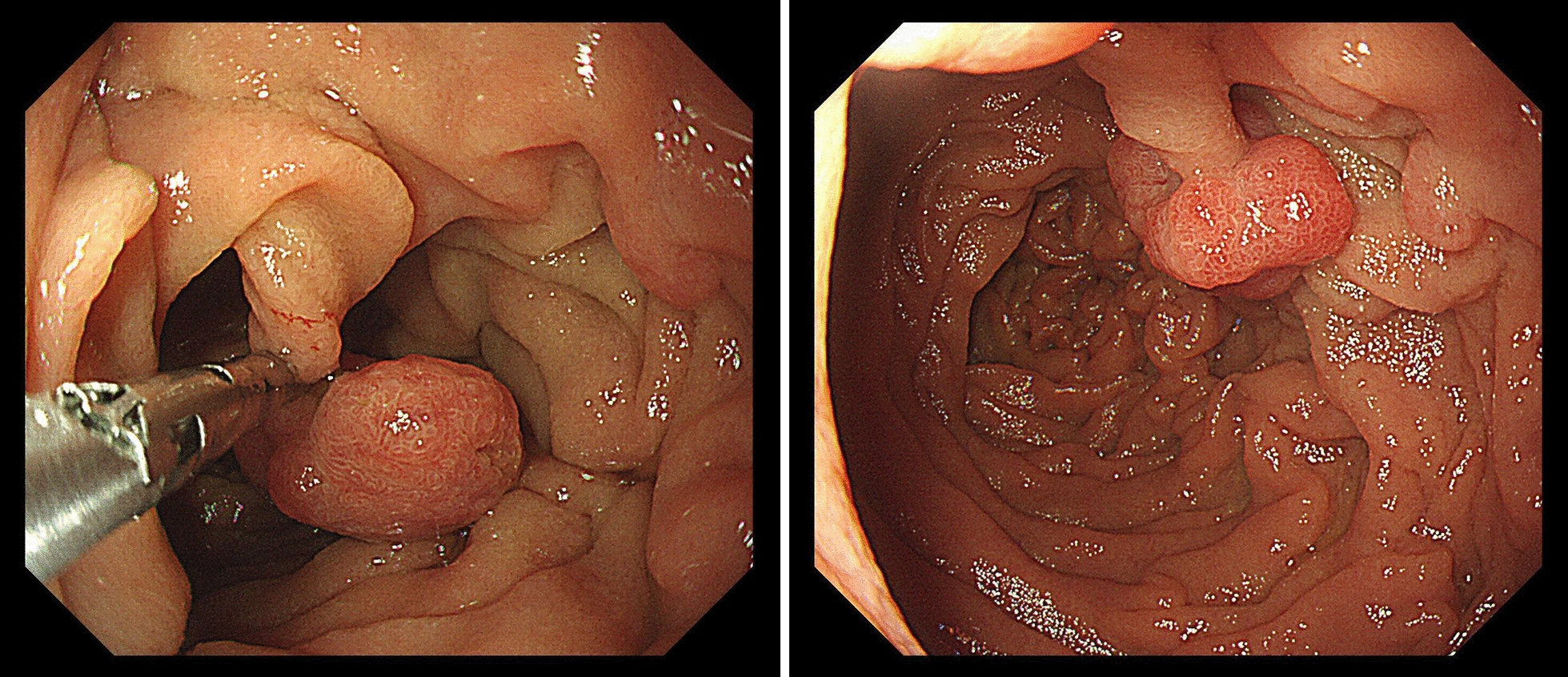
Peutz-Jeghers syndrome polyps. A 42-year-old female PJS patient being treated with ischaemic polypectomy

PJS causes characteristic mucocutaneous pigmentation and intestinal (predominantly small bowel) hamartomatous polyps (Fig. 4) and disease pathophysiology is not fully established [93]. Dysplasia in polyps is rarely observed, and it is likely that PJS polyps are not pre-malignant [94]. Small bowel polyps causing intussusception is the greatest risk in childhood. The cumulative intussusception risk is estimated at 50–68% during childhood and up to 30% of patients require surgery before age 10 [95, 96]. In adulthood, in addition to polyp-related complications, PJS confers an increased risk of cancer. The data are subject to significant selection bias, but the overall lifetime risk has been reported to be 55–85% [97–103], with the most commonly seen malignancies being breast and pancreatic cancer [94].

Current treatments for PJS include pan-enteric surveillance and polypectomy starting at 8 years of age, with a view to preventing polyp-related complications. Elective polypectomy via device-assisted enteroscopy or intraoperative enteroscopy should then be performed for small-bowel polyps if they are either > 15–20 mm in size, or are symptomatic [104]. Breast surveillance is recommended, but the role of surveillance for pancreatic and gynaecological cancers is not clear [104]. Inhibition of mTORC1 may represent an avenue for chemoprevention in PJS—a mouse model showed rapamycin treatment was sufficient to reduce polyp burden [105], and a single human report has demonstrated partial remission in advanced pancreatic cancer [106].

### Polyhydramnios, megalencephaly, and symptomatic epilepsy syndrome

Polyhydramnios, megalencephaly, and symptomatic epilepsy syndrome (PMSE or Pretzel syndrome) is an ultra-rare neurodevelopmental disorder caused by variants in the *LYK5/STRADA* gene. STE20-related kinase adaptor α (STRADA) is a pseudokinase that forms a heterotrimeric complex with LKB1 and the scaffold protein calcium binding protein 39 (CAB39, also known as MO25) [107]. STRADA activates LKB1 through an allosteric mechanism leading to phosphorylation of LKB1 (Fig. 2) [26].

PMSE is characterized by severe, infantile-onset intractable epilepsy, developmental delay, macrocephaly, craniofacial dysmorphism and premature mortality [108]. Homozygous single nucleotide variants and larger deletions including exons 9–13 in the *LYK5/STRADA* gene are associated with the STRADA phenotype. Pre-clinical models are available [109, 110] and a human cortical organoid model suggested that megalencephaly arises from expansion of neural stem cells in early corticogenesis and potentially also from increased outer radial glia at later stages [111]. In a small clinical study sirolimus treatment reduced seizure frequency in PMSE patients [109].

### Tuberous sclerosis complex

Tuberous Sclerosis Complex (TSC) has an estimated incidence of between around 1 in 6000 and 1 in 13,000 live births worldwide and across all ethnic groups [112–115]. Inherited in an autosomal dominant manner, approximately 70% of affected individuals have no family history of the condition and harbour a de novo pathogenic variant [116]. TSC is caused by loss-of-function variants in either of two genes, *TSC1* or *TSC2* [117, 118]. TSC1 and TSC2 form a complex, together with TBC1D7, that inhibits mTORC1 activity through the GAP activity of TSC2 towards RHEB (Fig. 2) [119]. The mutational spectrum in TSC is broad and includes deletions, nonsense and missense variants. Patients with causative variants in *TSC2* usually present with a more severe phenotype than those with *TSC1* variants, characterized by a higher number of tubers, earlier age at seizure onset and higher prevalence of intellectual disability [120]. Otherwise, the clinical phenotypes are highly variable and there is little genotype/phenotype correlation [121, 122]; the exception to this being a contiguous deletion on chromosome 16 that encompasses both *PKD1* and *TSC2* resulting in a compound phenotype of polycystic kidney disease together with TSC [123]. TSC demonstrates extreme inter- and intrafamilial variability [122].

TSC is characterised by the development of benign hamartomas affecting different organs at different stages of life and including the brain, heart, kidneys, skin and lungs [116, 124, 125]. The most comprehensive understanding of the clinical presentation was obtained from international patient registry data; and informs best practice for clinical surveillance and treatment of the complex co-morbidities of TSC [126].

Antenatally, cardiac rhabdomyomas may be identified on foetal ultrasound after 20 weeks gestation in TSC patients [127]. Typically, these undergo spontaneous regression postnatally and rarely require intervention [128, 129].

Skin changes may be observed from the neonatal period onwards, including hypopigmented macules, which are often the first presenting feature of the condition postnatally, shagreen patches, facial angiofibromas, forehead plaques and subungual fibromas [130]. Moreover, UV damage of the second copy of *TSC1* or *TSC2* is sufficient to cause angiofibromas [131].

**Fig. 5:**
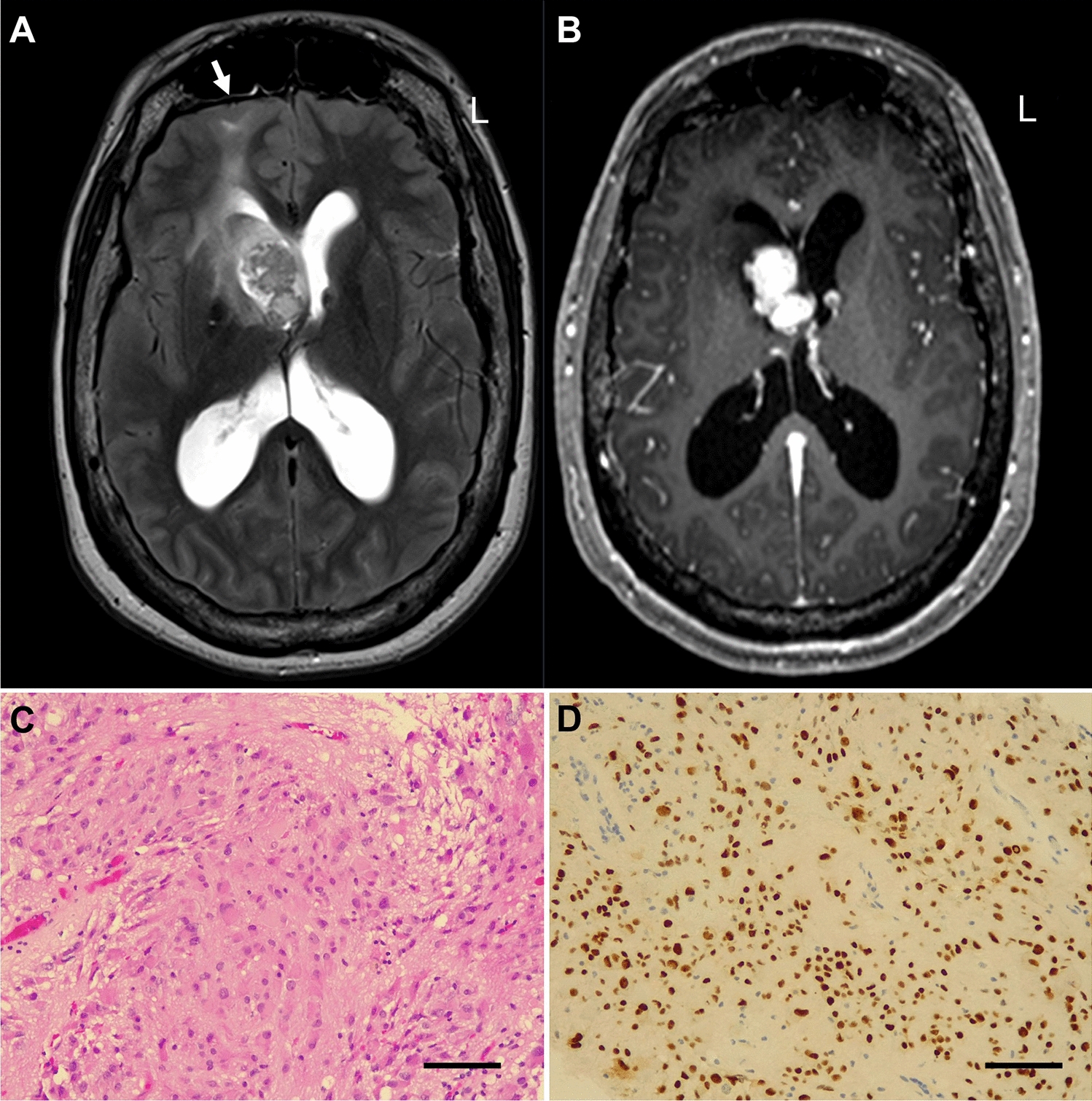
Neurological manifestations of TSC. 34-year-old female TSC patient with a germline TSC1 null mutation (Pro266ArgfsTer5). T2w (**A**) and postcontrast T1w (**B**) MRI images showing a large enhancing mass (SEGA) at the foramen of Monro with obstructive hydrocephalus and surrounding oedema. Background multiple cortical tubers were present, one of which is seen in the right frontal lobe (white arrow). **C** Histology of subependymal giant cell astrocytoma showing of spindle and epithelioid cells with abundant eosinophilic cytoplasm. **D** Immunohistochemistry for thyroid transcription factor 1 (TTF-1) displays expression in the nuclei. Bar: 50 mm

Epilepsy occurs in around 85% of affected individuals and in most, the onset of seizures occurs in the first two years of life, often with associated developmental regression and encephalopathy [132]. Initial presentation is often with infantile spasms and evolution to multi-focal seizures related in part to the number and location of focal cortical dysplasias (cortical tubers) that are typically seen in the brain of affected individuals (Fig. 5); the seizures are often refractory, with limited response to conventional anti-seizure medications [133]. Up to 24% individuals with TSC develop sub-ependymal giant cell astrocytomas (SEGAs; Fig. 5), which may need surgical or medical intervention if they enlarge [125, 134, 135]. TSC is also associated with a wide range of neurodevelopmental and psychiatric disorders including developmental delay, intellectual disability and ASD, psychiatric disorders, neuropsychological deficits, school and occupational difficulties—collectively known as TSC-Associated Neuropsychiatric Disorders (TAND) [136–138].

Kidney involvement occurs in about 80% of patients and is characterised by the development of angiomyolipomas (AMLs) and cystic kidney disease from childhood onwards [139]. AMLs that grow to greater than 3 cm in diameter are associated with a risk of life-threatening haemorrhage. Renal cell carcinoma is a rare complication of TSC, occurring in around 1–3% of TSC patients but with a striking female preponderance [140, 141]. TSC is relatively infrequently associated with progressive chronic kidney disease, with around 1–10% of patients developing end stage kidney disease [142, 143].

Traditionally treatment of TSC has focussed on management of symptoms, including antiseizure medications for epilepsy and embolization for large kidney AMLs that are at risk of bleeding. However, clinical trials of mTOR inhibitors demonstrated regression of tumours in the brain and kidney and stabilisation of lung function in those patients with lymphangioleiomyomatosis (LAM) [144–146]. These drugs are now licensed for use in the management of kidney AMLs, pulmonary LAM, SEGA, facial angiofibromata and refractory epilepsy in the context of TSC. Temporary use of mTOR inhibitors for cardiac rhabdomyomas that are impacting on cardiac function and are inoperable can be considered; but there remains paucity of evidence [147].

### TBC1D7-associated neurodevelopmental disorder

TBC1D7 binds to TSC1 to maintain the integrity of the TSC1-TSC2 complex (Fig. 2). Biallelic homozygous pathogenic variants in *TBC1D7* were identified in the children of consanguineous parents from two separate families, consistent with autosomal recessive inheritance [148, 149]. The phenotype included macrocephaly and mild intellectual disability. Head size was enlarged at birth and remained greater than the 98th centile. Functional studies demonstrated loss of expression on TBC1D7 and an increase in mTORC1 pathway activity. A further patient was identified in a study of patients with undiagnosed neurodevelopmental disorders who had compound heterozygous variants in *TBC1D7*, again consistent with a loss-of-function disease mechanism [150]. Schrotter et al., reported the generation of a *TBC1D7* knock-out mouse in which they demonstrated increased mTORC1 signalling and identified brain overgrowth due to thickening of the cerebral cortex, a phenotype consistent with that found in humans [151]. The paucity of reports suggests that variants in *TBC1D7* are a very rare cause of macrocephaly and intellectual disability.

### RHEB-associated neurodevelopmental disorder

RHEB belongs to a superfamily of small GTPase proteins [152], is highly conserved and activates mTORC1 through direct interaction (Fig. 2). Of the two RHEB proteins, RHEB (a.k.a RHEB1) and RHEB like 1 (RHEBL1; a.k.a RHEB2), found in mammalian cells, the ubiquitously expressed RHEB is required for the mid-stage embryonic viability and myelination of the brain, whilst RHEBL1 was found not to be essential for mTORC1 or mTORC2 signalling, or myelination [153].

Three cases with de novo germline hyperactivating *RHEB* variants have been described: two were siblings (suggesting parental gonadal mosaicism) and all had a heterozygous missense variant in the Ras effector domain of the protein [154]. All three individuals presented with early brain overgrowth and macrocephaly (+ 2.5/ + 3 SD) in childhood and had short stature (− 2 to − SD). They had severe to profound intellectual disability and ASD. Two had epilepsy, the third had epileptiform discharges on EEG but no seizures. Expression of these hyperactivating *Rheb* mutations in vivo in zebrafish (*Danio rerio*) and mice recapitulated migrational defects, increase in neuronal soma size, macrocephaly and seizures. These phenotypes were rescued by treatment with rapamycin, supporting the hypothesis of a dominant gain-of-function effect of these *Rheb* mutations [154].

Current treatments for patients affected by RHEB-related epilepsy include escalation of antiseizure medication and epilepsy surgery for focal cortical dysplasia type IIA/B (FCDII; see below). In hemimegalencephaly or megalencephaly, a surgical procedure called hemispherotomy or functional hemispherectomy is required to control seizures. This involves the surgical disconnection of one region or hemisphere, whilst leaving the disconnected brain within the skull. In other cases, an anatomical hemispherectomy is required. Surgical outcomes vary depending on whether there is contralateral involvement but can achieve a seizure-free rate of up to 50–60% of patients and significant improvement in seizure control in another 10–15% (reviewed in [155]).

### Smith-Kingsmore syndrome

Germline gain-of-function *MTOR* variants cause Smith-Kingsmore syndrome (SKS). SKS is an autosomal dominant, ultrarare neurodevelopmental disorder characterised by variable learning disability in combination with (prenatal onset) megalencephaly (head circumference is generally at least + 2 SD) [156–158]. Additional SKS clinical features include ASD, brain MRI anomalies (particularly cortical dysplasia), afebrile seizures, sleep disturbance, vascular abnormalities and hypotonia [159].

A hotspot variant, targeting amino acid residue 1799 (NM_004958.4: c. 5395G > A p.(Gly1799Lys), is currently the most common SKS variant [160, 161]. This variant is also an established somatic driver variant, identified in a range of different cancers [162]. Other missense variants, with functional data supporting causality in SKS, include Cys1483Phe [156]; Phe1888Cys and Met2327Ile [163].

Although data are limited, there is currently no evidence that individuals with SKS are at an increased risk of developing cancer and there are no SKS-specific established cancer screening programmes.

Whilst there has been considerable interest in the efficacy of mTOR inhibitors in SKS, there are currently no published data to support their widespread use. This is, in part, because SKS is ultrarare and, in part, because quantification of a response to treatment is challenging where impact on cognition and behaviour is being monitored.

### Birt-Hogg-Dubé syndrome

Birt-Hogg-Dubé (BHD) syndrome is a rare inherited genetic disorder with an estimated prevalence of 1 in 200,000 [164, 165]. Over 500 families with BHD have been reported world-wide. *FLCN* is the causative gene of BHD and functions as a tumour suppressor that regulates cell growth, energy/nutrient homeostasis, metabolism and autophagy (reviewed in [166]). BHD predisposes patients to benign skin lesions called fibrofolliculomas that form from hair follicles on the face, neck and chest [167]. BHD patients are also at risk of developing slow growing kidney tumours after 30 years of age and in less than a third of patients. Hybrid oncocytic renal tumours are characteristic to BHD [168]. The recommendation is that these tumours are monitored yearly, and kidney neoplasms greater than 3 cm in diameter are surgically removed. At the time of surgery, smaller neoplasms are typically removed to avoid further surgery later in life. If left, these tumours can progress into renal cell carcinoma (management of disease can be found here [169]). This 3 cm rule is considered an appropriate measure of when to remove these neoplasms by surgery. However more recently, a renal tumour less than 3 cm was found to be metastatic, indicating that tumour size might not be a reliable readout for some BHD patients. This study highlights a clinical need for better diagnostic markers of disease progression in BHD. Another key feature of BHD is lung cysts, where patients are at risk of spontaneous lung collapse (pneumothorax) that may need treatment to allow the lungs to re-inflate by needle aspiration or chest tube insertion [167].

FLCN is thought to play a central role in orchestrating homeostatic balance of energy and nutrients within cells as they grow. To do this, FLCN associates with large protein complexes on the surface of lysosomal membranes that regulate mTORC1 (Fig. 2) [31]. The Rag-Ragulator complex on the membrane surface of lysosomes has been progressively elucidated over the last few decades and shows direct association of FLCN to this complex (reviewed in [166]). Loss-of-function mutations in FLCN impair energy and nutrient sensing and signal transduction at lysosomes causing dysregulated mTORC1 signalling. *FLCN*-deficient cells then lose homeostatic balance and become metabolically challenged, likely the main driver of tumour growth. In a kidney specific *FLCN* knockdown mouse model, mTOR inhibition with rapamycin was found to reduce the enlarged size of the polycystic kidneys that developed [170]. More recently, it was uncovered that loss of *FLCN* alters mTORC1-directed phosphorylation of downstream substrates to favour TFEB phosphorylation [171]. This results in constitutive activation of TFEB (a master regulator of lysosomal biogenesis and autophagy) and is considered to promote disease features associated with BHD. Supporting this, in a kidney-specific BHD mouse model, it was shown that TFEB depletion rescued cysts formation and enlargement of the kidneys [171], suggesting that mTORC1 hyperactivity towards TFEB is a central driver of BHD. While major advances have been made in the understanding of FLCN at the molecular level, no specific treatment for BHD patients is currently available.

### GATORopathies

GATOR1 possesses GAP activity towards the Rag small G proteins that inhibit mTORC1 (Fig. 2), thereby repressing cell growth. GATOR1 is composed of the subunits Dishevelled, Egl-10, Pleckstrin domain-containing protein 5 (DEPDC5), nitrogen permease regulator-like 2 and 3 (NPRL2, NPRL3). Heterozygous pathogenic variants in *DEPDC5, NPRL2* and *NPRL3* are a major cause of focal epilepsy and are collectively classified as “GATORopathies”, with a prevalence of 0.2–3% for deleterious *DEPDC5* variants in large international collaborative studies [172, 173]. *DEPDC5* variants account for most GATOR1-related epilepsies (83%), possibly due to the greater length of the *DEPDC5* transcript [174]. Loss-of-function variants account for 60–70% of mutations and result in loss of inhibition of mTORC1. The remaining 30% of the mutations are missense variants, more commonly associated with non-lesional epilepsies [175] and not associated with mTORC1 hyperactivation.

GATORopathies display incomplete penetrance, interfamilial variability and predominant central nervous system phenotypic expression (reviewed in [176]): up to 60% are dominantly inherited from seizure-free parents, and present as a broad spectrum of lesional and non-lesional focal epilepsies, ranging from the paradigmatic familial focal epilepsy with variable foci (FFEVF) to frontal and temporal lobe epilepsies and epilepsy with centrotemporal spikes. No distinct phenotypic characteristics distinguish individuals with *DEPDC5, NPRL2* and *NPRL3* mutations. The age-of-onset ranges from childhood/adolescence to older than 50 years, about half experience seizures out of sleep and the majority suffer from drug resistant epilepsy [174, 177]. Individuals with lesional GATORopathies have been reported as having malformations of cortical development (MCD), including bottom of sulcus dysplasia (BOSD), FCD type I and II, hemimegalencephaly (HME), polymicrogyria, and subcortical band heterotopia, with FCDII as the most common MCD. Associated cognitive and psychiatric comorbidities are common, with 40% affected by psychiatric disorders, 9% having comorbid ASD and recent reports of ASD only phenotypes. Patients carrying GATOR1 subcomplex mutations are at increased risk of sudden unexpected death in epilepsy (SUDEP), although further studies are required to elucidate whether GATOR1 complex mutations directly influences SUDEP (e.g. through alteration of cardiovascular expression of DEPDC5 and NPRL3) or whether the increased prevalence of SUDEP in GATORopathies simply reflects the severity of the epilepsy expression (174, 178–181).

Favourable epilepsy surgical outcomes have been reported in patients with GATOR-related epilepsies who underwent resective surgery (where 80% of patients achieved a good surgical outcome, Engel I or II) and modern surgical techniques such as laser interstitial thermal ablation allow removal of multiple epileptic foci and have already shown promise in a case of epilepsy with variable foci due to an *NPRL3* mutation [182].

mTOR inhibitors have shown efficacy in GATORopathies involving a loss-of-function mechanisms in both preclinical models [176] and rare human cases [183] and represent a promising therapeutic strategy to lower seizure frequency and reduce SUDEP risk in patients who are unsuitable or have failed resective surgery.

### KPTN-related disorder

*KPTN*-related disorder is an autosomal recessive disorder associated with germline variants in *KPTN* (previously known as kaptin), a component of the mTOR regulatory complex KICSTOR that also includes SZT2, ITFG2 and KICS2 (Fig. 2) [184]. *KPTN*-related disorder is rare, and its prevalence unknown. The disorder was originally delineated in the Ohio Anabaptist (Amish and Mennonite) communities in 2014 [184]. To date, 61 individuals from 32 families (including 16 of Anabaptist heritage) have been identified associated with 20 biallelic pathogenic or likely pathogenic *KPTN* variants.

**Fig. 6:**
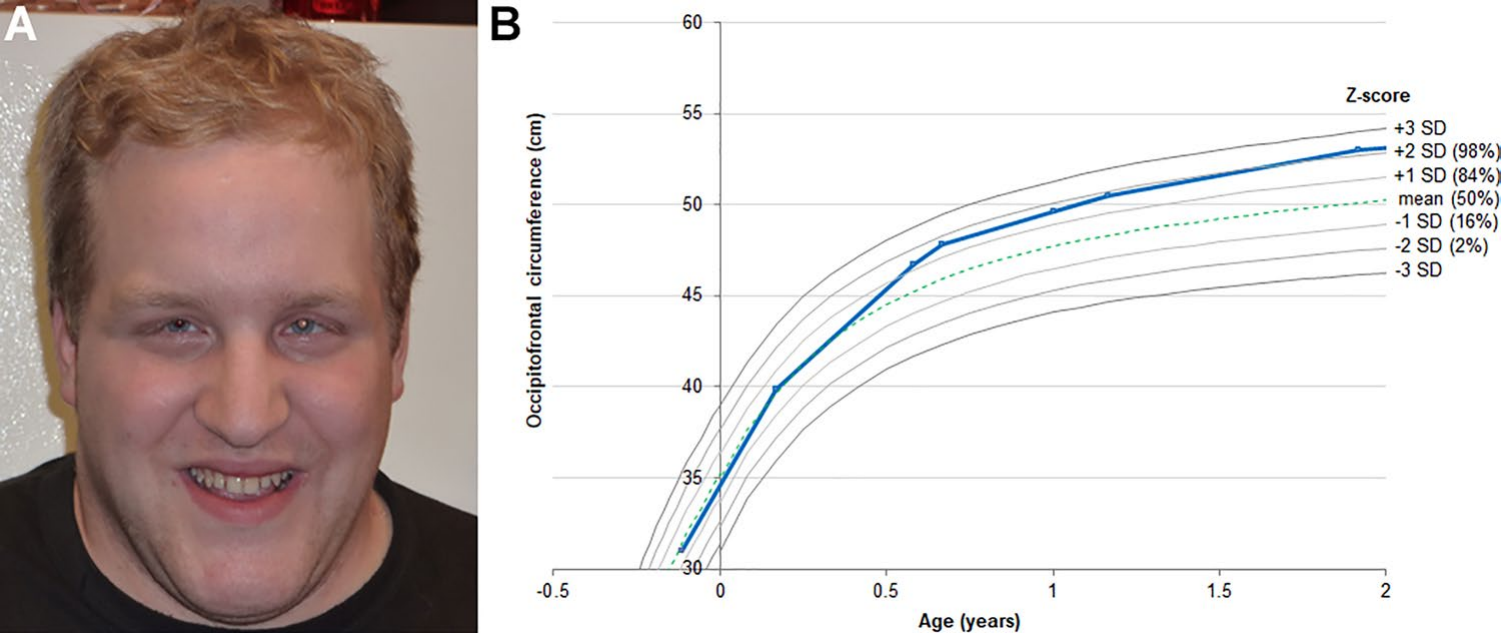
Clinical manifestations of KPTN-related disorder. **A** Craniofacial appearance of a 19-year-old male with KPTN-related disorder, a macrocephalic neurodevelopmental disorder with subtle facial dysmorphology, including frontal bossing, a prominent chin, small downslanting palpebral fissures and a broad nasal tip. Reproduced from [184]. **B** Occipitofrontal circumference in centimeters for a male individual with KPTN-related disorder. OFC increased from below the 50th centile (mean) at birth to over two standard deviations (SD) above the mean by the age of two years (blue line). Centiles given in brackets. Reproduced from [190]

The condition is characterised by global developmental delay, hypotonia in infancy, mild-to-profound intellectual disability, neurobehavioural/psychiatric manifestations (including anxiety and findings associated with ASD such as stereotypies, hyperactivity, repetitive speech, and impaired social communication), characteristic craniofacial appearance (frontal bossing, long face with prominent chin, broad nasal tip and hooded eyelids) (Fig. 6A), seizures and post-natal progressive macrocephaly with occipitofrontal head circumference (OFC) measurements up to + 6.1 SDs in adulthood. Neuroimaging findings typically comprise of a globally enlarged brain structure with otherwise normal morphology. Other more variable features include recurrent upper and lower respiratory tract infections, conductive hearing impairment, strabismus, nystagmus, ketotic hypoglycemia, thyroid dysfunction, early puberty, mild skeletal manifestations, hepatomegaly and splenomegaly [184–190].

OFC measurements are usually within normal limits at birth, but unlike other megalencephaly syndromes, OFC rapidly increases over the first two years of life (Fig. 6B), with ~ 70% of affected adults having an OFC > 2 SDs. Although *KPTN* heterozygous carriers are clinically unaffected, OFC measurements are increased when compared to population OFC. Around half of affected individuals develop seizures, either generalized tonic–clonic seizures alone, or with absence and/or complex partial seizures. Seizure onset can occur at any age and seizures are often refractory to polytherapy, with no anti-seizure medications identified as having greater efficacy [190].

*Kptn* knockout (KO) mice show increased mTOR signalling in the brain downstream of Kptn that is rapamycin sensitive and display many of the key *KPTN*-related disorder phenotypes, including brain overgrowth, behavioural abnormalities, and cognitive deficits. Differentiated human induced pluripotent stem cell models of the disorder also display transcriptional and biochemical evidence for altered mTOR pathway signalling, supporting the role of KPTN in regulating mTORC1 [190].

Interestingly, biallelic variants in *SZT2* also cause a distinct but similar rare neurodevelopmental disorder characterised by severe early-onset epileptic encephalopathy, global developmental delay, structural brain abnormalities such as thick and short corpus callosum and macrocephaly [191, 192]. *Szt2* KO mice display epileptogenesis and increased mTOR signalling in the brain [193, 194].

## Rare genetic diseases caused by somatic mutations in mTOR pathway genes

### Focal cortical dysplasia type IIA/B, hemimegalencephaly and megalencephaly

**Fig. 7:**
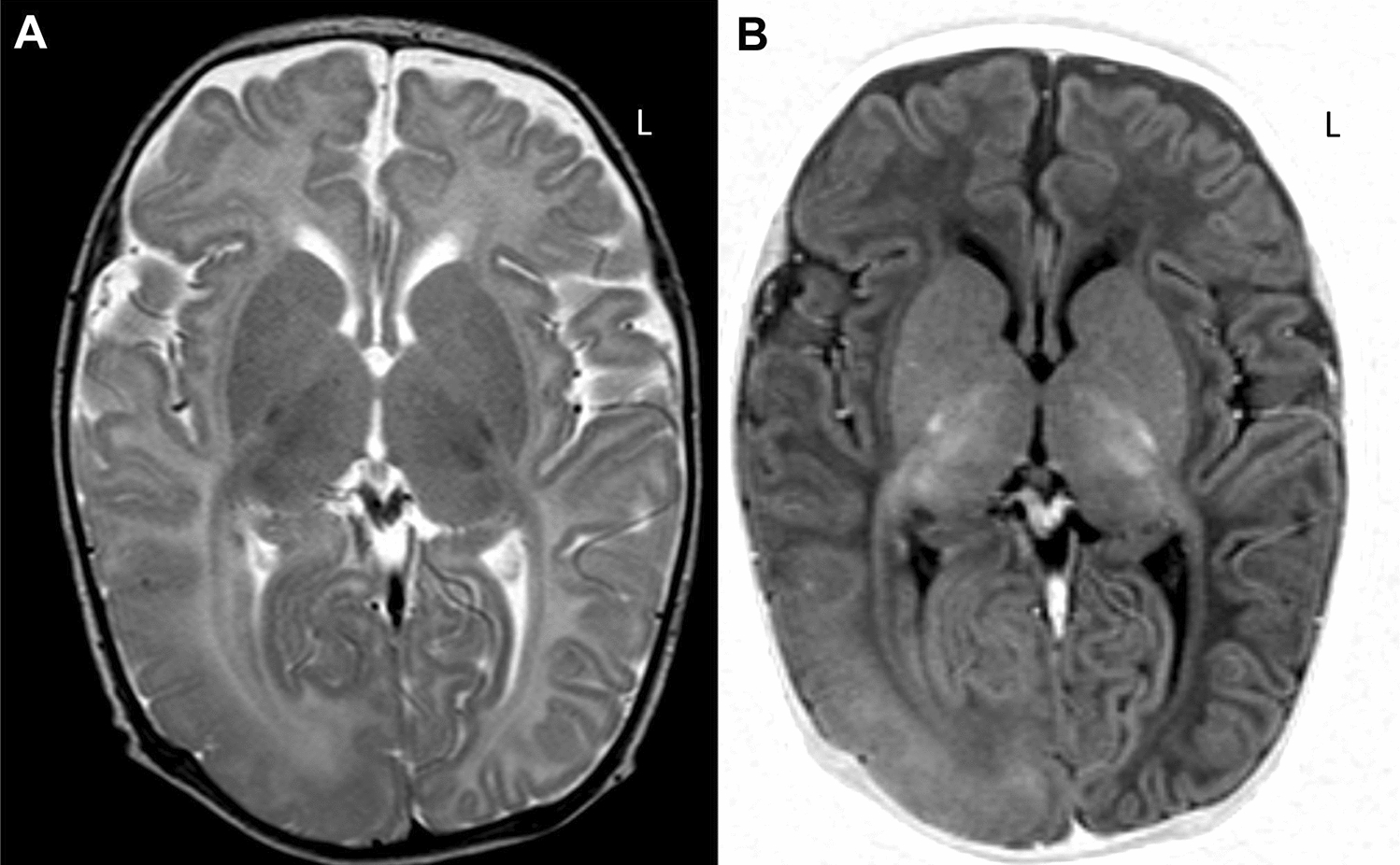
Hemimegalencephaly MRI. MRI from a female at age 2 months. **A**, **B** T2w and T2w images showing enlargement of the right occipital and adjacent posterior temporal/parietal lobes with diffuse thickening of the cortex, abnormal shallow overlying sulcation and underlying white matter signal abnormality. Findings are consistent with posterior quadrantic dysplasia (localised or hemi hemimegalencephaly)

**Fig. 8:**
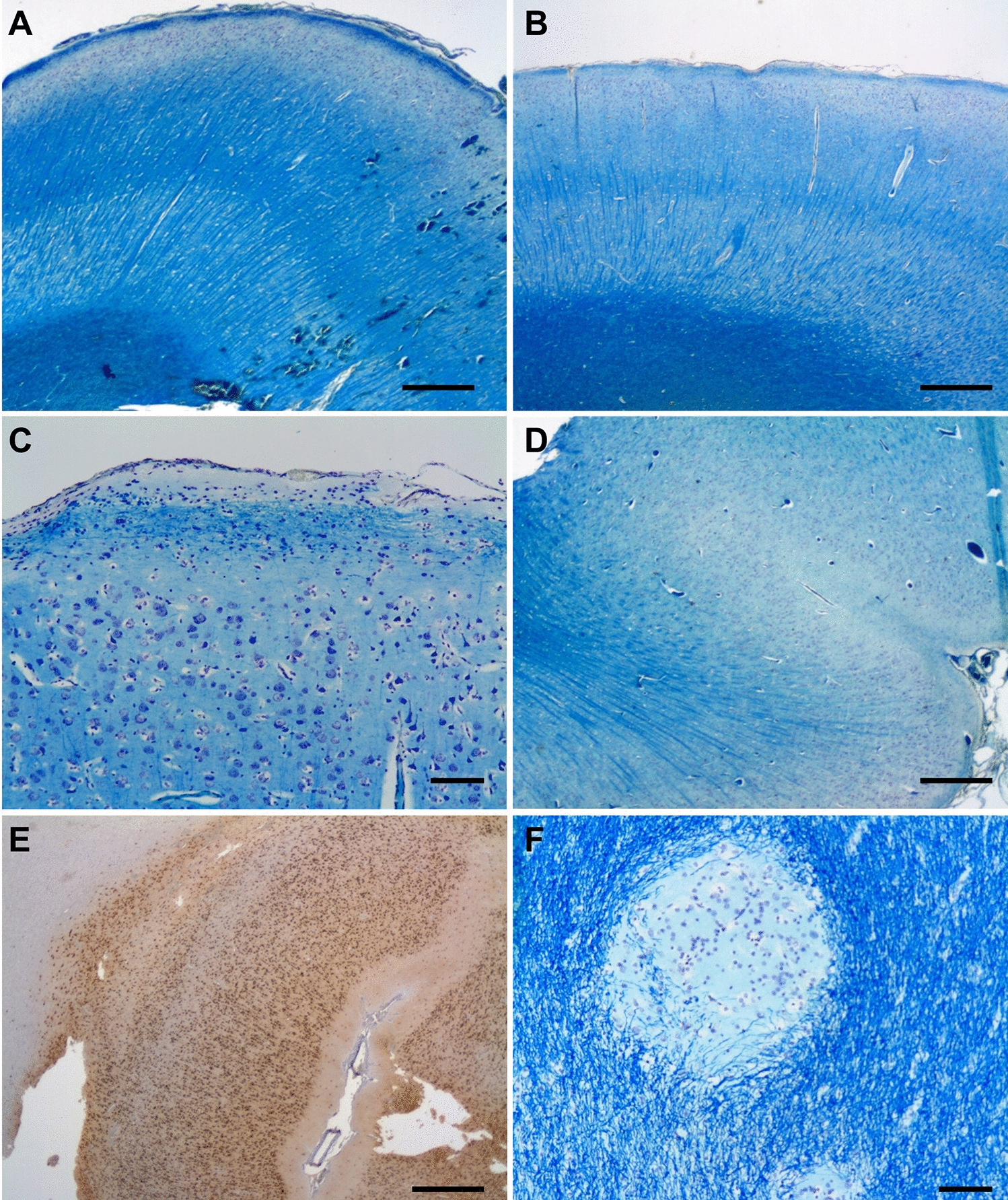
Hemimegalencephaly histology. **A**, **B** Complex histological abnormalities in posterior quadrantic dysplasia (localised or hemi hemimegalencephaly) (Luxol fast blue-Nissl staining). The cortical lamination is abnormal with myelinated fibre layer in the middle of the cortex. The neurons are almost exclusively pyramidal cells with absence of granular cell layers. **C** The lamina 1 contains increased number of cells, occasionally pyramidal cells, and in areas parallel running myelinated fibres are seen. **D** In places the grey and white matter demarcation is blurred due to large neurons splaying into the subcortical white matter, immunohistochemistry for neuronal nuclear protein N—NeuN). **E** Immunohistochemistry for NeuN. **F** Occasional the heterotopic nodules are also noted. (Bars A, B, D, E: 1 mm; Bars C, F: 100 µm)

**Fig. 9:**
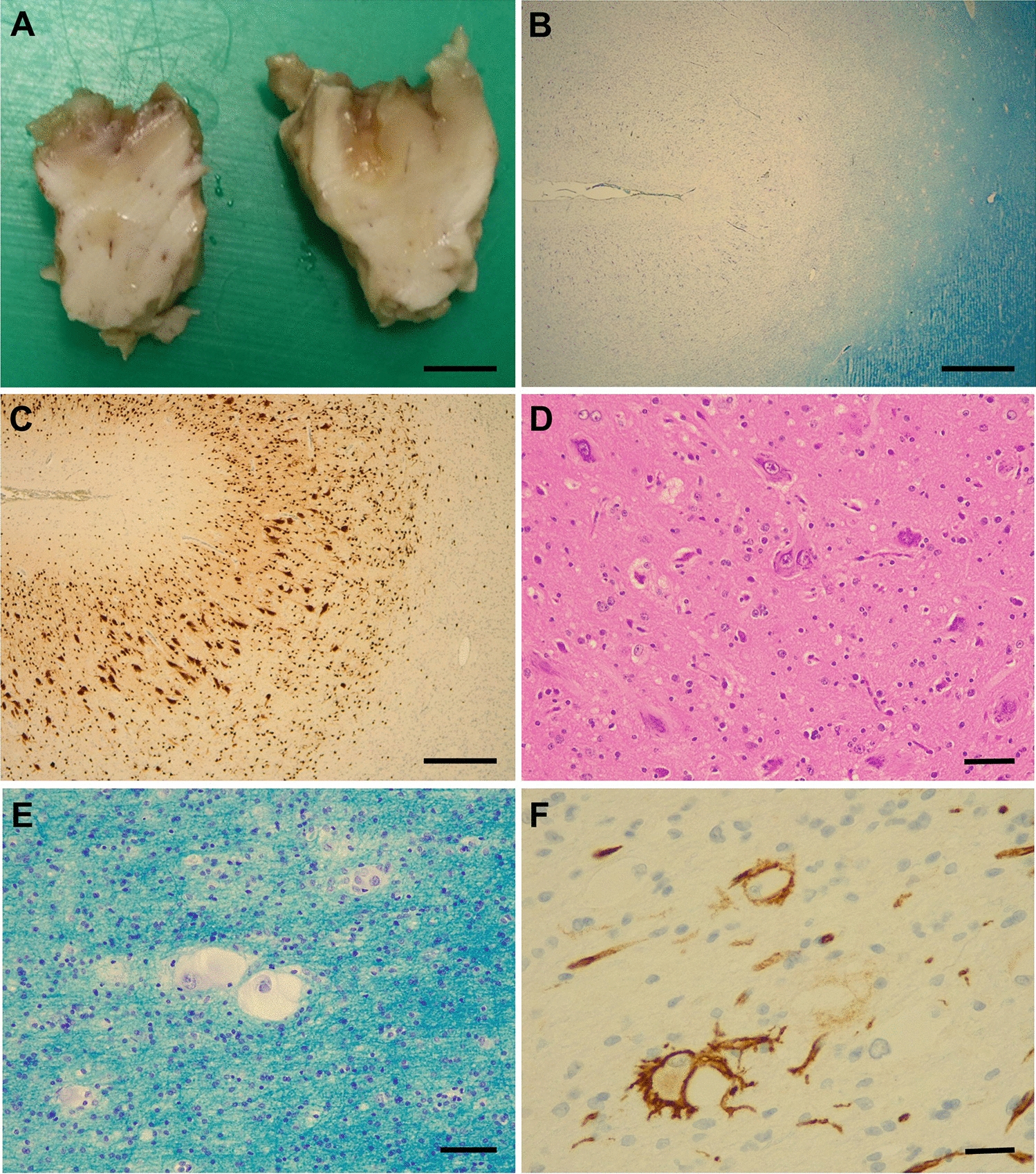
Focal cortical dysplasia (FCD) type IIb. **A** Slicing of the lesion after formalin fixation reveals blurred demarcation between cortex and white matter, confirmed by Luxol fast blue-Nissl staining (LFB-N) (**B**), also showing neurons splaying into the white matter. **C** Dyslamination of the cortex with presence of large (dysmorphic) neurons by immunohistochemistry for neuronal nuclear protein N (NeuN). **D** The dysmorphic neurons have abnormal orientation and frequently show chromatolysis (haematoxylin & eosin staining). **E** The ballooned cells are most common in the subcortical white matter (LFB-N). **F** Immunohistochemistry for CD34 may label glial cells with bushy ramified processes around some of the balloon cells. Bar A: 1 cm, Bars B, C: 1 mm; Bars D-F: 100 mm

Postzygotic (or somatic) mutations in mTOR-pathway genes from human brain tissue were first shown to be a major cause of large cortical malformations (HME and megalencephaly ME; Figs. 7, 8), and later also identified in smaller cortical malformations (focal cortical dysplasia type IIA/B (FCDIIA/B), BOSD, where they arise in neural progenitor cells at a later stage of development and cause a clonal population of mutated cells (Fig. 8) (reviewed in [195–197]). The net effect of pathogenic variants is to lead to hyperactivation of mTORC1, either via a gain-of-function heterozygous variant in *MTOR* or upstream activators (*AKT3, PIK3CA, RHEB)*, or in rare cases via a double-hit inactivating mutation in a repressor gene (*DEPDC5, TSC1/2 and NPRL3*) (Fig. 2) [198]. In FCD, pathogenic variants are found in dysmorphic neurons or balloon cells (only FCDIIB) (Fig. 9), whilst in HME/ME glial cells also carry mutations, implying these arose in earlier progenitor cells during development. The size of the cortical malformation is directly related to the timing of the mutational hit and the variant load: in 80% of FCDIIA/B individuals, less than 5% of cortical cells carry a pathogenic variant (corresponding to a variant allele frequency of < 2.5%) and high read depth sequencing (at least 1000X) is required to improve diagnostic yield. In HME/ME variant allele frequency is higher, with up to 60% of cells carrying a pathogenic variant [195].

Cortical malformations are associated with medically intractable focal epilepsy arising from childhood to early adulthood, with FCD-related epilepsies representing 9% of the epilepsy surgical population [199]. Surgical resection or thermal ablation of FCDs is associated with favourable outcomes (Engel I and II in 70–80% of patients) in drug refractory cases (reviewed in [177]). Within the dysplastic area, the region with the highest variant load is associated with maximal epileptogenicity [200, 201]: detection of the mutation gradient within the epileptogenic zone, e.g., through testing depth electrode-derived tissue [202] and removal of the area with the highest mutation load may improve surgical outcomes.

Whether mTOR inhibitors are safe and effective in patients with FCD has been addressed in individual case reports [203, 204], two completed small trials (14 patients in [205], 16 patients in [206]) and one open label trial [207]. Whilst mTOR inhibitors were deemed well tolerated, reduction in seizures in children with FCD did not reach the predefined level of statistical significance in the open-label trials and only one patient underwent genetic testing and had a confirmed mosaic *MTOR* variant [203]. Further studies are necessary to identify novel diagnostic techniques to increase presurgical diagnostic yield of genetic testing, and those patients who would benefit most from mTOR inhibition.

### PIK3CA-related overgrowth spectrum

**Fig. 10:**
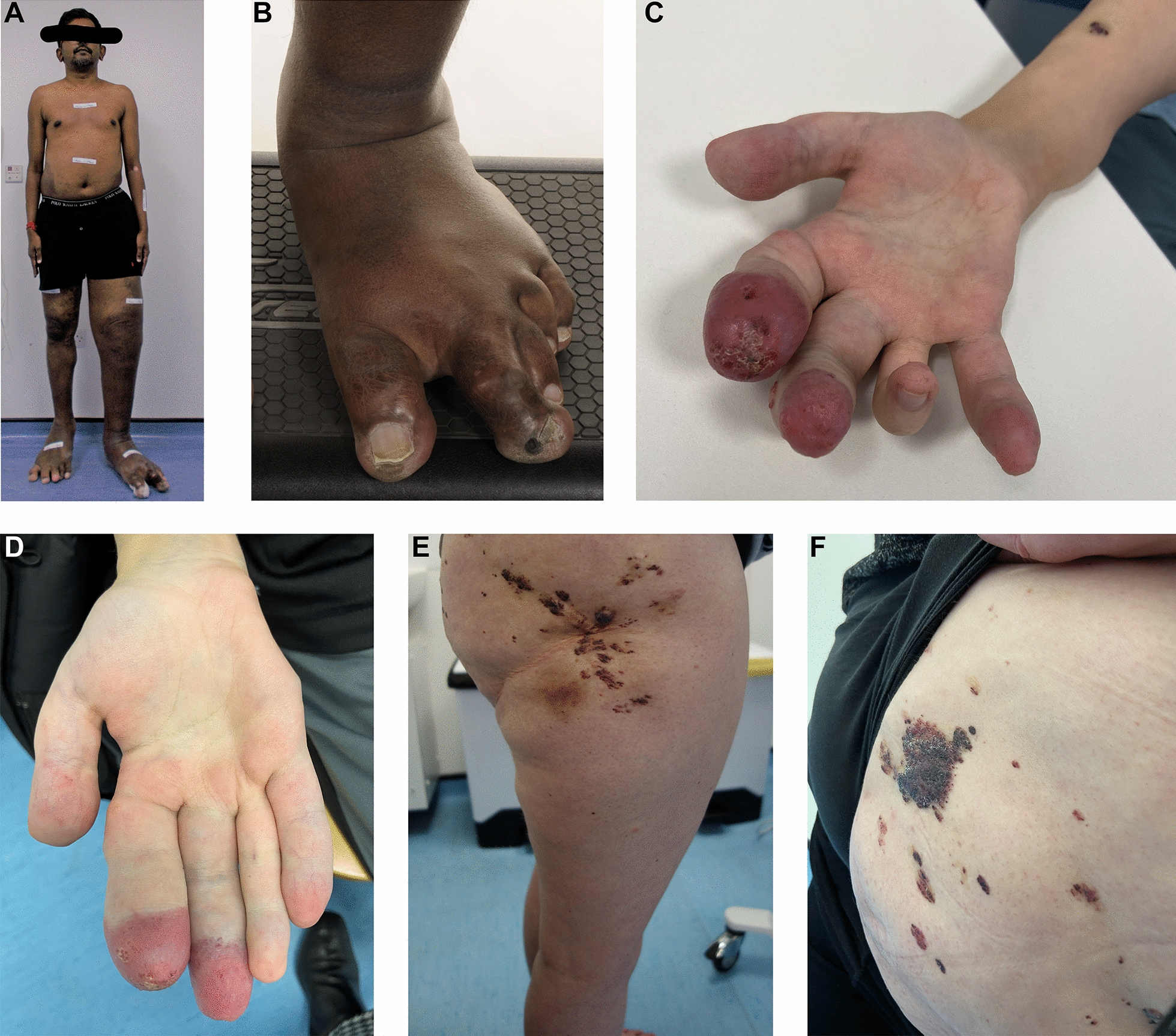
Clinical manifestations of PROS. **A**, **B** A 44-year-old male with CLOVES—congenital lipomatous malformation of the trunk, segmental overgrowth of both legs, extensive vascular and venous malformations, and scoliosis. There is macrodactyly of the toes on both feet with typical appearance (wide sandal gap and relatively short hallux). Skin biopsy of affected tissue identified a pathogenic variant in PIK3CA (c.1039-1041AAA. P.Val346_347ins Lys) in 50% of cells. **C**, **D** Extensive venous malformations of the right hand with leaking lymphangiectactic blisters at the tips of the index and middle finger and macrodactyly. Skin biopsy of the affected tissue identified a gain-of-function pathogenic variant in PIK3CA (c.1633G > A. P.Glu545Lys) in 13% of cells. **E**, **F** A 40-year-old female with a lympho-veno-vascular malformation of the right thigh and buttock and segmental overgrowth of the right leg (diagnosed as Klippel–Trénaunay syndrome). Current problems with pain and leakage from lymphangiectatic blisters (seen in the images). Skin biopsy identified a pathogenic variant in PIK3CA c.3140A > G. p.His1047arg in 4% of cells

Post-zygotic mosaic (somatic) activating pathogenic variants in *PIK3CA* may result in diverse developmental disorders depending on the embryological timing of the variant, the tissues and site involved, the type of pathogenic variant and the mosaic ‘load’ of the activating pathogenic variant. These conditions have been grouped under the umbrella term ‘*PIK3CA*- Related Overgrowth Spectrum’ (PROS) [208]. The clinical presentation may be as minimal as macrodactyly of a single digit or as complex as CLOVES (Congenital Lipomatous Overgrowth, Vascular malformations, Epidermal naevi, Spinal/skeletal anomalies/scoliosis) or MCAP (Megalencephaly-CAPillary malformation) (Fig. 10). PROS is associated with multiple complications and disabilities including progressive overgrowth, increased risk of infections (cellulitis) and venous malformation with an increased risk of superficial and deep thrombosis. The malformations are often present at birth but may increase/evolve over time [209, 210].

In view of the broad spectrum of presentation it is very difficult to assess the prevalence of these conditions. One recent paper cited an incidence of 1 in 27,000 [211].

The management of this cohort is mainly supportive but there is an increasing role for mTOR inhibitors, e.g., sirolimus, which can have a moderating effect on the progression of the disease [212, 213]. Moreover, clinical trials using agents that specifically target PI3K (e.g. alpelisib) are underway for PROS, with some positive results to date [214, 215].

### Lymphangioleiomyomatosis

Loss-of-function mutations in *TSC1* or *TSC2* cause LAM, a systemic disease causing lung cysts and lymphatic abnormalities [216]. LAM occurs as a rare sporadic disease but also affects over half of adult women with TSC [217]. The disease is strikingly female-specific, with the sporadic form appearing completely restricted to women. The overall prevalence of LAM is around 20 per million women, with no known ethnic or racial predominance [218, 219]. Sporadic LAM results from sequential somatic mutations in *TSC2*, whereas TSC-LAM is the result of germline *TSC1/2* mutations followed by loss of heterozygosity with mutations spread across the whole gene [220, 221]. Both *TSC1* or *TSC2* mutations cause TSC-LAM, although LAM is more common and more severe in those with *TSC2* mutations [222]. In all cases, loss-of-function of the TSC1/TSC2/TBC1D7 complex results in activation of mTORC1 and phosphorylation of downstream substrates including p70S6K and 4E-BP1 [119, 223]. These mutations occur in a yet unknown mesenchymal cell which, as a result of mTOR dysregulation, clonally expands and acquires a metastatic phenotype with a tropism for the lungs and axial lymphatics, whilst mTORC1-driven glycolytic (Warburg) metabolism allows the LAM cells to survive in these environments [224].

**Fig. 11:**
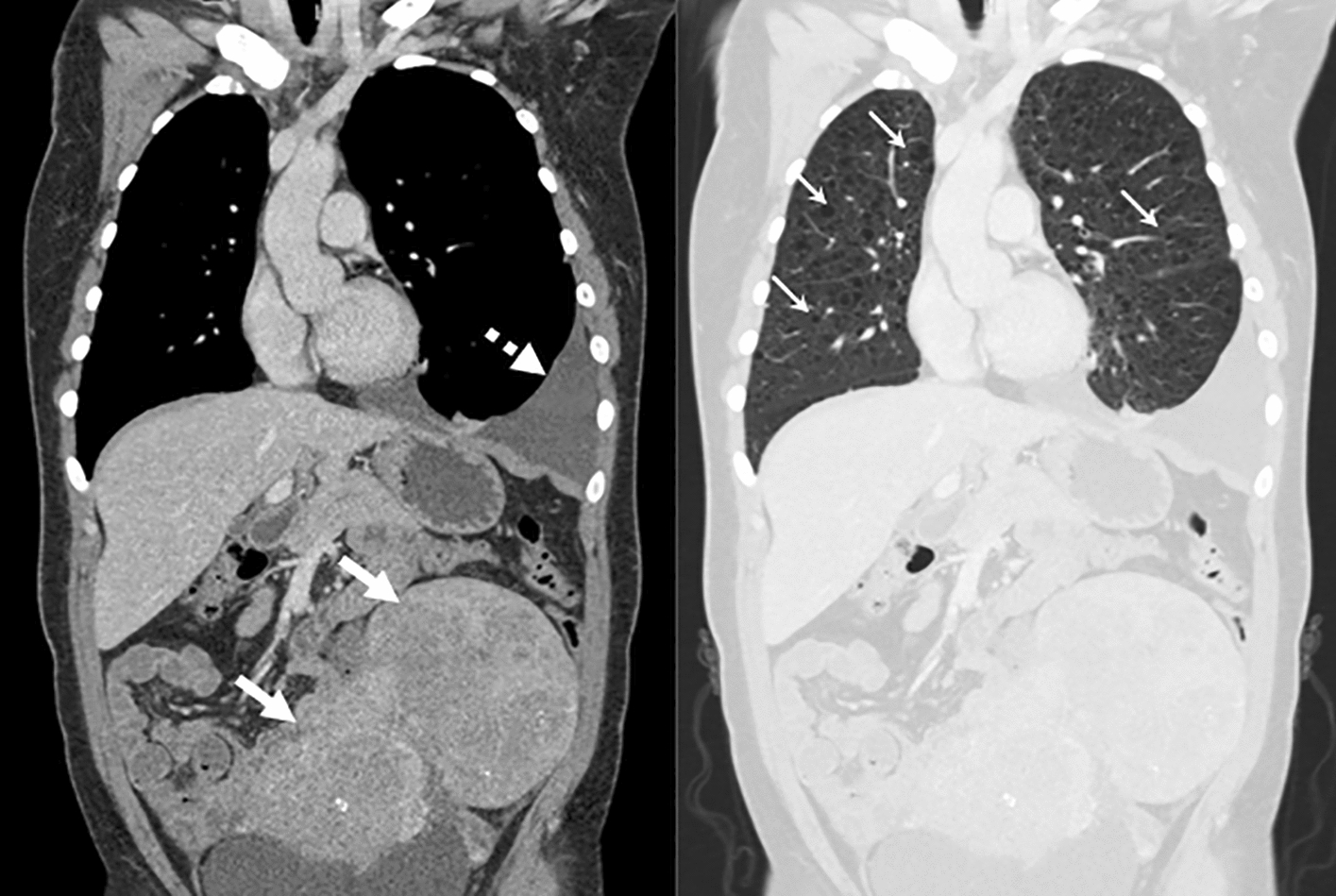
Clinical manifestations of LAM. Coronal CT scan of the chest and abdomen of a patient with LAM. Soft tissue windows (left) show a large, complex abdominal lymphangioleiomyoma (white arrows) and a left sided chylous pleural effusion (dashed arrow). Lung windows (right) show multiple air-filled lung cysts distributed throughout the lung parenchyma (some highlighted by small arrows)

LAM cells, likely by chemokine production, attract fibroblasts, alveolar type 2 cells, inflammatory and lymphatic endothelial cells to form LAM nodules within the lung [225–229]. LAM nodules, probably by protease secretion, form cysts causing recurrent pneumothorax, progressive airflow obstruction and impairment of ventilation perfusion matching [230–232]. Patients experience a loss of forced expired volume in 1 s (FEV_1_) of around 120 mL/year; over six times faster than healthy people [233]. Lymphatic obstruction causes chylous collections in the abdomen and thorax in around 20% of patients (Fig. 11) [216].

LAM is incurable, but the mTOR inhibitor rapamycin reduces lung function loss and is highly effective for chylous collections and other aspects of TSC [234, 235]. However, the drug does not completely suppress disease activity, possibly due to non-mTOR dysregulated wild-type cells in LAM nodules [236, 237]. Airflow obstruction can be treated with bronchodilators, with supplementary oxygen and pulmonary transplantation sometimes required [216].

### Proteus syndrome

Proteus syndrome is a rare overgrowth syndrome (~ 200 reported cases worldwide) caused by a gain-of-function somatic mutation (c.49G > A, p.Glu17Lys) in the AKT serine/threonine kinase 1 (*AKT1*) gene [238]. The net effect of the mutation results in AKT1 activation, leading to activation of mTORC1 signalling, promoting growth and limiting apoptosis in nearly every region of the body [239]. Starting in infancy, proteus syndrome patients present with asymmetric overgrowth, connective tissue nevi, epidermal nevi, cranial hyperostosis, visceral hamartomas, and vascular anomalies. A cerebriform connective tissue nevus (CCTN) of the soles may be a specific finding but is not pathognomonic. The disease is progressive and results in death [240] by compression or distortion of vital structures, thromboembolism, malignant transformation of overgrowing tissue or respiratory disease from pulmonary cysts [241]. Intellectual disability and seizures may be the presenting features in individuals with the less common central nervous system manifestations of Proteus syndrome, including hemimegalencephaly, cerebral arteriovenous malformations, abnormal grey-white matter differentiation, neuronal migration disorders, callosal dysgenesis, and hydrocephalus [242, 243].

Until recently, only palliative treatment was available for individuals with Proteus syndrome. Recently, miransertib (ARQ 092) an oral, allosteric, selective pan-AKT inhibitor was identified, trialled in patients with ovarian cancers bearing an activating AKT1 variant and reported showing favourable efficacy and side effects [244, 245]. Furthermore, six Proteus syndrome patients were treated with 5 mg/m^2^/day of miransertib (1/7th oncologic dose) in a pilot study, which was well tolerated and achieved the primary endpoint of a 50% reduction in the tissue levels of AKT phosphorylation from biopsies in 5/6 affected individuals, as well as a decrease in a cerebriform connective tissue nevus and a reduction in pain in children [246]. An adult man was subsequently reported being treated on a compassionate scheme with miransertib titrated up to 25 mg/m^2^/day for one year, resulting in improved general well-being, increased mobility, reduced CCTN and a whole-body MRI showing no apparent disease progression [241].

## Current mechanism-targeting treatments for mTOR pathway diseases

### Everolimus and sirolimus for TSC and LAM

Clinical guidelines align well with the clinical trial evidence supporting the use of everolimus and sirolimus in TSC and LAM [125]. The use of everolimus in TSC is supported by a range of clinical trials. The EXIST-1 trial, a phase III, randomized, placebo-controlled trial demonstrated that everolimus significantly reduced the size of SEGAs in individuals with TSC [247]. Response rates (≥ 50% reduction in target volume relative to baseline) were higher in the everolimus group compared to placebo (35% vs. 0%). The EXIST-2 trial showed that everolimus significantly reduced the volume of kidney AMLs compared to placebo. The AML response rate (≥ 50% reduction in total volume of target AML relative to baseline) was significantly higher in the everolimus group (42% vs. 0%) [248]. The EXIST-3 trial evaluated the efficacy and safety of everolimus in patients with TSC who have drug-resistant epilepsy. Everolimus significantly reduced seizure frequency in TSC patients with drug-resistant epilepsy, with both high-exposure and low-exposure groups showing benefit over placebo. However, the beneficial effects do not continue after treatment ends. The treatment was generally well-tolerated, with a manageable safety profile [249]. Everolimus is approved for treating SEGAs, kidney AMLs and TSC-related refractory epilepsy by both FDA and EMA. There are no strong guidelines for its use in LAM, and it is used off-label based on limited evidence.

Sirolimus has demonstrated efficacy in reducing SEGA volume in TSC patients in a non-randomized, open-label study [250]. It is used off-label for TSC-related tumours when everolimus is not suitable or available.

Sirolimus is FDA, EMA and MHRA approved for the treatment of LAM. The MILES trial, a phase III, randomized, placebo-controlled trial demonstrated that sirolimus stabilizes lung function decline in individuals with LAM. Patients receiving sirolimus had a significantly slower rate of decline in FEV_1_ compared to placebo [146]. The use of sirolimus is recommended for LAM patients with abnormal or declining lung function, chylous effusions, or symptomatic LAM [251].

Topical rapamycin is an effective treatment option for facial angiofibroma in TSC [252]. A preparation of topical sirolimus marketed as Hyftor is now approved by the FDA, EMA and MHRA, although funding for prescription on the NHS has yet to be approved.

### Alpelisib for PROS

By the time *PIK3CA* variants were identified as the genetic cause of PROS, targeted PI3K inhibitors were already advancing in oncology [253]. Hopes were therefore high that such therapies could eventually be repurposed as effective treatments for PROS. In 2018, this seemed within reach after an initial report of substantial benefit to 19 PROS patients, including two with life-threatening disease, upon treatment with the PI3Kα-selective inhibitor alpelisib/Piqray® [254]. This was an unregistered index study, however, and subsequent follow-up studies have highlighted the need for caution and, most importantly, a randomised controlled trial (RCT). A retrospective study (EPIK-P1) of patients with severe or life-threatening PROS with managed access to alpelisib reported that 38% of 32 studied individuals presented with a measurable reduction in “target lesion” volume and improvements in other symptoms; however, 39% of the study population suffered from alpelisib-related adverse effects [214]. Such adverse effects are unsurprising given the importance of PI3Kα for normal cell function. Moreover, although alpelisib acts preferentially on PI3Kα, at clinically relevant doses it is also likely to inhibit PI3Kβ/δ/γ [255]. This may contribute to some of the reported adverse effects, especially those involving the immune system. Uncertainty therefore remains regarding alpelisib’s therapeutic window, safety and overall efficacy in PROS where life-long treatment is likely to be required [256]. Reflecting this uncertainty, EMA recently highlighted lack of long-term safety data, particularly in relation to growth and development, as one of the major objections precluding conditional marketing authorisation of alpelisib for PROS [257]. By contrast, the results from EPIK-P1 were sufficient for accelerated FDA approval of alpelisib for the treatment of adults and children (2 years of age or older) with severe PROS [258]. Ultimately, however, a comprehensive and unbiased evaluation of alpelisib in PROS will only emerge once the ongoing RCT, EPIK-P2 (NCT04589650), is completed in 2031.

### Leniolisib for APDS

The characterisation of the genetic defect of APDS has allowed the repurposing of PI3K inhibitors previously used in haematological malignancies (reviewed in [255]). Initial clinical trials, however, were complicated by considerable toxicities (hepatitis, colitis, pneumonitis) and infection susceptibility [259]. In 2014 idelalisib became the first FDA-approved PI3Kdelta inhibitor licensed to treat relapsed chronic lymphatic leukaemia as a third line-treatment for follicular lymphoma and small lymphocytic lymphoma [255]. Leniolisib, a more selective PI3Kdelta inhibitor, was repurposed and proven effective to treat APDS [50, 260]. This new, targeted therapy was granted orphan drug designation status by the EMA in late 2021 and is currently undergoing evaluation by NICE in the UK. Medium term safety and tolerability appears favourable [261], but vigilance regarding any impact on lymphoma predisposition will be essential [75].

### Drugs in development for mTOR pathway diseases

Development of mTOR inhibitors has rapidly progressed since the discovery of rapamycin and other allosteric mTOR inhibitor rapalogs for clinical use (sirolimus, everolimus, temsirolimus) [262]. Second generation mTOR inhibitors, including torin-1, torin-2 and vistusertib, are ATP competitive mTOR kinase domain inhibitors, competing with ATP for the catalytic site, which target both mTORC1 and mTORC2. Third generation mTOR inhibitors, RapaLink 1 and 2, combine the allosteric mTORC1 specificity of rapamycin with an mTOR kinase domain inhibitor [263]. An ingenious strategy has also been developed using a brain impermeable analogue of FK506 known as RapaBlock, to restrict the activity of RapaLink 1 to the brain [264]. Despite these advances in the development of second and third generation mTOR inhibitors, they have yet to be used in clinical trials for mTOR pathway diseases.

A number of drug trials for mTOR pathway diseases are currently ongoing. Metformin activates AMPK resulting in inhibition of mTORC1 [265]. Metformin treatment reduced SEGA volume and seizure frequency in a small randomised double-blind placebo-controlled trial in TSC [266]. More recently, the NIHR released a commissioned call to address whether metformin is effective in reducing seizures in TSC [267].

In PHTS trials of mTOR inhibitors (sirolimus, everolimus) are reported to be tolerated and have some efficacy in treating cutaneous and gastrointestinal features and improving cerebellar function, as well as in treatment of vascular anomalies [79–81]. A trial using sirolimus for treatment of colon polyps for Cowden syndrome is ongoing (ClinicalTrials.gov ID: NCT04094675). Improvement in some measures, but not reaching primary endpoint was demonstrated in a trial of treatment of everolimus for neurocognitive symptoms in PHTS [79]. Two patients with germline PTEN mutations and breast cancer showed a dramatic response to treatment with the AKT inhibitor Capivasertib [82].

There is significant use of mTOR inhibitors off label in the treatment of mTOR pathway diseases. In the UK, sirolimus is currently under consideration by NHS Clinical Commissioning as an intervention for extracranial slow-flow vascular malformations that are refractory to standard therapies in PROS. An open-label observational study was performed in five patients with drug-resistant epilepsy caused by variants in the GATOR1 complex genes *DEPDC5* and *NPRL3*. All four patients with *DEPDC5* variants showed reduced seizure frequency, while seizures worsened in the *NPRL3* patient [268].

## Challenges and future opportunities in mTOR pathway diseases

### Underlying mechanisms

Since the discovery of mTOR in the 1990s, research into this kinase, the complexes it forms, and its regulatory mechanisms has transformed the way we understand fundamental processes in cell biology. However, although we have an exceptional knowledge of the function of the core components of the pathway, the role and function of the downstream targets is much less understood. Whilst a handful of mTORC1 substrates (S6K, 4E-BP, ULK1, and TFEB), are well characterised, we have little understanding of the detailed regulatory mechanisms of the vast majority of direct and indirect mTORC1 targets. Phospho-proteomic studies in mammalian cell lines identified 85–174 proteins whose phosphorylation was regulated by mTOR [269–271]. A recent survey identified 56 and 26 *bona fide* substrates of mTORC1 and mTORC2 respectively [272]. Gene editing now provides the means to precisely mutate phosphosites in mTOR substrates to test their functional role in biology and their potential contibution to mTOR pathway diseases.

In the context of human disease, mTOR signalling touches upon nearly all aspects of medicine including immune disorders, cancer, metabolic disorders, neurodegeneration and neurodevelopmental diseases [11]. The major challenge now is to exploit our detailed knowledge of the molecular and cellular mechanisms of this pathway to improve understanding and provide new treatments for human disease. As a group, mTOR pathway diseases affect most tissues and organs and even individual diseases can affect multiple tissues. We still have very little idea of why specific tissues are affected in diseases that are associated with the mTOR pathway. Recent technologies such as phospho-proteomics and single cell sequencing can be applied to analyse patient tissue and sophisticated disease models utilized to identify common and divergent targets, including potential biomarkers, that will provide deep insight the enigma of tissue specificity.

Cellular and animal models of mTOR pathway diseases, by design, produce highly reproducible phenotypes within and between experiments. This markedly contrasts with the clinical manifestations of mTOR pathway diseases, which are highly variable, often making diagnostic criteria hard to define. Despite a common underlying molecular mechanism, the clinical presentation across mTOR pathway diseases is diverse, affecting the brain, kidney, lung, skin and haematopoietic system and this phenotypic heterogeneity is currently unexplained. Within individual mTOR pathway diseases there is also an unexplained broad clinical spectrum. In TSC for example, patients with, in some cases the exact same, loss-of-function mutations in *TSC2* can have symptoms ranging from severe epilepsy starting in infancy to mild skin lesions [121, 124]. There is also little genotype–phenotype correlation between *TSC* gene variants and disease severity [121, 273]. This phenotypic heterogeneity presents major challenges to accurate prognoses and predicting the efficacy of existing drugs. The identification of genetic modifier loci in cystic fibrosis (CF) has shown not only that these contribute to the phenotypic heterogeneity in CF but that modifiers can be organ specific. Moreover, data integration approaches with CF-specific biospecimens has led to the identification of novel therapeutic targets that have now been the subject of small molecule screens to develop modifier-directed therapies that are in pre-clinical studies [274–277]. Combined with well-defined patient populations, the rapidly falling cost of whole genome sequencing provides the opportunity to identify the genetic modifiers responsible for the broad heterogeneity in mTOR pathway diseases and utilise this knowledge to improve prognosis and develop precision medicine treatments.

mTOR pathway diseases associated with malformations of cortical development and intractable epilepsy, known as mTORopathies, cause the greatest morbidity and challenge to treatment [278]. Insight into the neurodevelopmental complexities in mTORopathies has been provided by mouse models [279, 280]. Conditional knockout of mTOR pathway genes has been complemented by in utero electroporation methods to model the genesis of cortical malformations in mTORopathies. CRISPR has recently been employed to inactivate *Depdc5*, *Tsc1* and *Pten* during mouse cortical development and directly compare the effects on cortical excitatory synaptic activity [281]. iPSCs and organoid models are also emerging as powerful tools to investigate the alterations in neurodevelopment in mTORopathies directly in human cells, reviewed in [12].

### Diagnosis and prognosis

#### Establishing a molecular diagnosis

Although diagnostic criteria are well established for mTOR pathway diseases, and targeted genetic testing or gene panel testing by whole genome sequencing (WGS) is available in England [282], primary clinical diagnosis is not always straightforward. Due to the significant phenotypic heterogeneity patients often present to a range of different clinical specialists and therefore require an astute physician to consider a genetic referral. Even when there is a clear clinical diagnosis, genetic testing may be negative; possible explanations include somatic mosaicism, complex structural variation, deep intronic variants or undiscovered genetic heterogeneity. For example, in those with a clinical diagnosis of TSC, pathogenic variants are identified using standard technology in around 85% of patients, while somatic mosaicism for pathogenic variants in *TSC1* or *TSC2* occurs in at least 10% of patients [283]. Access to deep sequencing technology in clinical care has proved highly successful in improving the diagnostic yield, offering more families the potential for more tailored offspring risks and prenatal or preimplantation genetic diagnosis [283].

In some health systems genetic testing may be unavailable because of funding or geographical issues [284], therefore depriving individuals of an accurate diagnosis and reproductive choice. Centralisation of funding for molecular testing in the UK has enabled more individuals to access testing in an equitable fashion [285].

Other challenges are presented when disease is caused by somatic mutations in inaccessible tissues, as in FCD; here diagnosis is probabilistic or confirmed after surgical resection and histological examination. Brain tissue can be hard to access for histological diagnosis without neurosurgical procedures. Sensitive, deep sequencing technologies may allow accurate detection of low frequency somatic variation from depth EEG electrodes when tissue is not resected. Less invasive approaches under investigation include high resolution neuroimaging combined with artificial intelligence to detect and classify subtle lesions.

#### Diagnostic biomarkers

As genetic testing is not always possible or successful or comprehensive, biomarkers detectable through non-invasive methods are another potential way to diagnose mTOR pathway diseases and disease manifestations. Liquid biopsies of peripheral blood or cerebrospinal fluid have promise. The best described example in the mTOR field is the use of serum VEGF-D as a marker for LAM diagnosis [286]. High serum VEGF-D levels can distinguish LAM patients from patients with other lung conditions, such as pulmonary Langerhans’-cell histiocytosis and emphysema [287]. A serum VEGF-D threshold of 800 pg/ml is the recommended cut-off for LAM diagnosis [251]. However, a low serum level cannot exclude a LAM diagnosis, while the utility of VEGF-D as a prognostic marker is unclear [251], so additional biomarkers are still needed. Analysis of extracellular vesicles (EV) in blood is another cutting-edge technique undergoing evaluation. For example, differences in plasma EV protein cargo are seen in TSC patient samples compared to healthy controls [288].

For those conditions with brain involvement, imaging and EEG markers can be useful in assessment. In the context of TSC, it was found that interictal fast ripples on scalp EEG could be a biomarker of epilepsy in children [289]. Circulating serum miRNAs and isomiRs have potential in the early risk assessment of ASD and intellectual disability development in TSC patients [290]. FCD is often not easy to diagnose and one study found MRI was unrevealing in a third of patients [291]. However, combining brain morphometric analyses such as voxel-based or surface-based morphometry to extract imaging biomarker information could improve the detection of FCD lesions that are undetectable by MRI [292]. Overall, while promising, there is still substantial work to be done on identifying diagnostic and prognostic biomarkers of mTOR pathway diseases.

#### Phenotypic variability and stratified care

Secondary diagnosis refers to the detection of the individual manifestations of disease once the primary diagnosis has been made. In common with many other rare diseases, mTOR pathway diseases show variability of expression and therefore all patients require screening for disease manifestations that they may never develop. Secondary diagnoses may not be evident at the time of presentation, for example the development of SEGA or TAND diagnoses [293] in children with TSC, or the growth of gastrointestinal polyps in PJS [93]. Currently, secondary diagnoses rely on serial monitoring and systematic screening, which may be expensive or invasive and challenging to perform in patients that are unable to cooperate with medical procedures.

Improving our understanding of phenotypic variability, for example cancer susceptibility in PHTS and PJS, or epilepsy risk in TSC, would lead to more personalised approaches to management and treatment. Susceptibility to secondary manifestations of disease might be revealed by large-scale genome-wide association studies, as exemplified in the autosomal recessive multi-organ disease CF [294], or the identification of biomarkers including radiological findings that predict the development or absence of complications. Given the phenotypic overlap between different mTOR pathway diseases, a specific mTOR pathway disease next generation sequencing panel in Genomics England National Genomic Test Directory would provide uniformity of access and diagnosis as well as an invaluable research resource.

#### Access to assessment and care

For families affected by mTOR pathway diseases, the multisystem nature of many of the conditions and the challenge of neurodevelopmental impairments is compounded by fragmented care [295]. Care during childhood is usually overseen by a coordinating paediatrician in the UK, but transition to adulthood is a vulnerable time as multidisciplinary care is lost [296]. Optimising early recognition and management of these conditions has the potential to reduce the burden of developmental issues and learning difficulties. This is particularly pertinent in families where both parents and children are affected. Improving the coordination of care is a key recommendation of the UK Rare Disease Framework but requires bold commissioning decisions in a complex health system. The development of rare disease clinics that provide access to multiple specialists as a 'one-stop shop' and access to clinical nurse specialists as points of contact may provide a solution to some of these difficulties [297].

#### Creating patient cohorts

Diagnosis also attains vital importance in epidemiology and public health when measured at the population level. In this context, individuals with a specific diagnosis need to be identifiable within health system records. Conventionally, this is achieved using diagnostic codes. Several coding systems pertain in different health systems. In the UK, World Health Organisation (WHO) International Statistical Classification of Diseases and Related Health Problems, Tenth Revision 5th Edition (ICD-10) codes are used in hospital care whilst the Systematised Nomenclature of Medicine Clinical Terms (SNOMED CT) is prevalent in primary care. ICD-10 codes have been assigned to only 500 rare diseases and this presents a problem in assessing burden and in patient identification or insurance reimbursement.

Coding representations to WHO may be made individually, although the process can be time-consuming and requires considerable resources [298]. A global approach is more powerful and efficient but some countries have tackled the issue at a national level. The Rare Disease Coding project (2013–2019) in Germany sought to address this by first, adding and assigning ICD-10-GM codes for diseases listed in OrphaNet missing in ICD-10; and second, assigning Alpha-IDs to synonymous terms of rare diseases mapped to the same OrphaNet and ICD-10-GM codes [299]. Once the coding problem has been solved, health records can be fully exploited for research and patient care.

There are still sparse data concerning epidemiology, natural history, treatment response, outcomes and health economics for most rare diseases. Importantly, knowledge about most of these data constitutes a fundamental starting point for industry partnerships, which are vital for progress in rare disease therapeutics. In England, the National Diseases Registration Service (NDRS) collects data on rare diseases under Section 254 of the Health and Social Care Act and so, importantly, does not require patient consent. Given sufficiently high-quality electronic patient records and coding, NDRS data could be used to generate unbiased estimates of epidemiology and comorbidity and to reveal inequalities or disparities in access or treatment. Linkage to associated population datasets could provide data on mortality, patterns of prescribing and health service utilisation. To date, several rare disease research projects have been supported by NDRS, e.g. [300, 301], and a population-based approach offers obvious advantages over clinically held databases or pharmaceutical sponsored registries. Population-based registries also offer the opportunity to investigate genetic and environmental modifiers underpinning phenotypic heterogeneity, leading to the development of precision medicine-based diagnostics and interventions. In the future, national registries could be a vehicle to identify geographically dispersed patients with specific characteristics for clinical trials.

#### Personalised medicine

Prediction of treatment response is another key area for advancement in rare diseases alongside diagnosis and prognosis. Using the example of TSC, not all patients that meet the criteria for the prescription of mTOR inhibitors will respond. The response rate in those with enlarging renal AMLs is high (around 97% in the EXIST-2 follow up [145]), but lower with epilepsy (50% long term reduction in seizures [302]). Better understanding of the factors that influence drug response and efficacy will facilitate a more personalised approach to prescribing.

### Development and approval of new therapies for mTOR pathway diseases

Knowledge around the genetics and neurobiology of neurodevelopmental disorders caused by mutations in genes in the mTOR pathway has expanded in recent years. However, for many mTOR pathway diseases there is no targeted treatment available, opening opportunities for development of new therapies to address the high unmet need. The development of new therapies is impeded by the slow, complicated processes that make drug development costly and the high attrition rates that create significant uncertainty regarding the return on investment [303]. Overall, rare, and ultra-rare diseases do not attract the same level of interest, research and investment as more common conditions. However, there is unique opportunity for a disease pathway approach by unifying multiple mTOR pathway diseases based on their common molecular mechanisms. This approach would provide several advantages by increasing critical mass through collaboration across different diseases and disciplines; reducing attrition due to genetically driven biology [304]; the possibility to derisk translation to clinic through the use of a rare disease model concept to arrive at proof of concept, and expansion of patient impact to additional mechanistically related diseases.

Drug repurposing offers a promising avenue, where existing drugs, both within and outside mTOR pathway indications, can be redirected to treat these rare conditions, potentially reducing development time and costs.

Working across diseases brings significant challenges related to the heterogeneity between these diseases that includes differences in unmet need, natural history of the disease and endpoints, including patient-centred outcomes. Initiatives involve establishing rare disease registries to facilitate the collection of essential information for designing clinical trials [305]. Dealing with this level of heterogeneity will require significant innovation with respect to trial design and regulatory flexibility, for instance when using a basket trial design approach (clinical trials including multiple diseases concurrently). Progress in the timely diagnosis of diseases and the development of biomarkers for patient stratification are essential to reconcile molecular biology with clinical endpoints. Currently, there are no non-invasive CNS mTOR pathway biomarkers that allow assessment of target engagement or early prediction of response in patients. Emerging technologies such as artificial intelligence and digital tools offer promising avenues to expedite and mitigate risks during clinical phases.

The authorization of over 240 orphan medicines in the EU over the past two decades [306] exemplifies the success of orphan drug pathways. Despite these early successes, we will need to continuously reflect and reimagine how to further incentivise development of innovative solutions for patients with rare diseases, including repurposing approved drugs for novel but mechanistically related conditions. Partnerships between academic institutions, patient advocacy groups, and pharmaceutical companies can facilitate the sharing of knowledge and resources, accelerating the path to new therapies.

## Integration of PPIE in mTOR pathway disease research

Integrating Patient and Public Involvement and Engagement (PPIE) from the earliest stages of research is recognised by the NIHR as crucial, ensuring research activities are carried out 'with' or 'by' members of the public or patients, rather than 'to' or 'about' them [307]. The aims of PPIE are broadly twofold: (1) to empower lay communities in an expert-dominated environment and ensure ethical medical research that addresses identified unmet needs and priorities; (2) to optimise the design, applicability and/or dissemination of the research itself, ultimately for more robust findings and effective interventions [308]. These aims are particularly pertinent for mTOR pathway disease communities presenting with unique challenges, including a limited patient population making it difficult to identify and involve a sufficient number of patients and representatives; diverse needs and perspectives between and within conditions; challenges analysing data; reduced resource and funding; and concerns around data security and confidentiality [309].

Broad-ranging benefits are expected from the integration of PPIE in mTOR pathway disease research. First, PPIE integration enhances potential to identify, recruit and retain participants, including those not yet diagnosed or without confirmed genetic diagnosis, to support clinical research and trials. The community are more likely to commit to research if they feel connected to and have supported the design of (for example, ensuring flexibility and support to travel to research sites). Second, effective PPIE fosters trust and transparency, crucial to addressing accessibility, data sharing, security and ethical concerns that may arise with establishing a patient registry. Third, involvement of patients and the public can improve the dissemination of research opportunities and findings, making the results more accessible and actionable for broader communities, with a higher likelihood of translation and adoption of research into everyday practice. Fourth, demonstrating robust PPIE can influence policy makers and funding bodies to support research initiatives, recognizing diverse stakeholder perspectives and inspiring further collaboration. Importantly, by integrating PPIE, we ensure the autonomy of people with lived experiences of mTOR pathway diseases is respected, enabling the community to influence research priorities and practice. Such efforts have the potential to enhance the relevance, dissemination and impact of research, ultimately leading to more patient-centred approaches and better outcomes.

## The mTOR pathway diseases node

The mTOR Pathway Diseases node [5] is one of the 11 nodes in the NIHR/MRC Rare Disease Research UK Platform (RDR UK) Platform [6]. RDR UK is a £14 M, 5-year project, beginning in 2023, and was established to connect and enhance the UK’s strengths in rare disease research. The platform fosters greater collaboration between academic, clinical and industry researchers, patients, research charities and other key organisations in rare disease research to accelerate the understanding, diagnosis and treatment of rare diseases. The mTOR Pathway Diseases node aims to unite rare individual mTOR pathway diseases as a single group based on a common underlying molecular mechanism: hyperactivation of the mTOR pathway. The node brings together clinicians, researchers, charities, industry and not-for-profit organisations to improve the diagnosis, treatment and clinical outcomes for mTOR pathway disease patients.

Research within the mTOR Pathway Diseases node targets many of the challenges and exploits the opportunities described in the preceding sections. These include: (1) building a comprehensive patient registry for all mTOR pathway diseases with the NDRS that will facilitate demographics and epidemiology, studies of drug responsiveness and provide natural history data for clinical trials; (2) building a tissue repository and analysing patients’ tissue using phosphoproteomics to identify new mTOR targets and give insight into tissue-specific mechanisms; (3) generation of mTORopathy patient-derived iPSCs with different responses to anti-seizure medications to reveal mechanisms underlying phenotypic heterogeneity.

PPIE is a key part of the mTOR Pathway Diseases node. Successful engagement from all mTOR pathway disease communities, including patient organisations and people with direct lived experience, is important to ensure proactive, balanced and proportionate representation and assessment of the impact of involvement. Incorporation of a variety of participatory active research methods, such as workshops with patient organisations, advisory panels of people with lived experience and governance roles on the steering committee, ensures diverse voices are heard.

The node has partnered with nine patient organisations representing mTOR pathway diseases. A PPIE advisory panel advises on all aspects of the node including study design, patient-facing materials, co-producing lay articles and media and co-authoring scientific papers. Effective PPIE practice in the mTOR Pathway Diseases node is inclusive (e.g. transparent PPIE recruitment process, accessibility of meetings), collaborative (e.g. co-produced terms of reference), supportive (e.g. identifying training needs on either side) and clear (e.g. regular and relevant communication) [310]. The higher prevalence of intellectual disability and neurodevelopmental conditions, such as ASD (see also Sect. “Diagnosis and prognosis”), necessitates a specialised approach to assessing care needs and promoting inclusivity in PPIE. In particular, patient organisations have direct access and insight into how to work effectively with patients and therefore can facilitate and encourage participation. Importantly, progress towards specific aims is documented and continuously evaluated with pre-defined assessment tools, to ensure prompt and effective problem-solving [308].

Examples of feedback from mTOR node PPIE advisory panel members:

“Involvement [with the mTOR node] enhances existing understanding of mTOR pathway in terms of biology and then the increased understanding of how this impacts the body and ultimately the individual with the condition or for the carer. This higher level of understanding provides a little comfort.”

“Effective involvement [with the mTOR node] enhances patient’s and carer’s existing understanding of the mTOR pathway and its implication in impacts on the body and ultimately the individual with the condition or their carer.”

“I feel personally this project [the mTOR node] doesn’t just empower but makes you feel you are doing something positive where you can feel alone and incapacitated. Such complex medical conditions can leave you feeling unable to affect positive change whereas being involved in a project such as this one [the mTOR node] gives you purpose. It is also important that it provides a voice for those who often do not have one.”

## Conclusions

mTOR pathway diseases are a subset of around 7000 rare and ultrarare diseases that affect 1 in 17 in the population, or over 3.5 million people in the UK alone. Rare disease research was for many years neglected but is now an energised and growing field. Recent funding of the RDR UK Platform and LifeArc Rare Disease Research Centres and similar international initiatives, including the European Joint Programme on Rare Diseases and the NIH Rare Diseases Clinical Research Networks, represent a step change in the rare disease field. The current challenges and opportunities in basic and clinical mTOR pathway disease research is a microcosm of the field overall. The future directions we have outlined here can therefore be applied to rare diseases in general.

## Data Availability

All relevant available data are included in the manuscript.
